# Cynarin Alleviates Sodium Iodate-Induced Retinal Pigment Epithelium Injury by Regulating Oxidative Stress and Inflammation

**DOI:** 10.3390/biom16091227

**Published:** 2026-08-24

**Authors:** Yue-Lin Fang, Yu-Jou Hsu, Chao-Hsien Sung, Chia-Chi Kung, Shiuan-Ruei Shiu, Chih-Yu Hung, Mei-Jung Chen, Der-Chen Chang, I-Chia Liang, Chi-Feng Hung

**Affiliations:** 1 PhD Program in Pharmaceutical Biotechnology, Fu Jen Catholic University, New Taipei City 242062, Taiwan; erickmichael2002@gmail.com (Y.-L.F.); s16179263@gmail.com (Y.-J.H.); a02664@mail.fjuh.fju.edu.tw (C.-H.S.); 2 Division of General Surgery, Department of Surgery, Shin Kong Wu Ho-Su Memorial Hospital, Taipei 111045, Taiwan; 3 Department of Anesthesiology, Fu Jen Catholic University Hospital, Fu Jen Catholic University, New Taipei City 24352, Taiwan; drpainkung@gmail.com; 4 School of Medicine, Fu Jen Catholic University, New Taipei City 242062, Taiwan; 5 Department of General Medicine, Chang Gung Memorial Hospital, Kweishan, Taoyuan 333423, Taiwan; hung1008@cgmh.org.tw; 6 Institute of Biomedical Engineering, School of Health and Medical Engineering, Ming Chuan University, Taoyuan 33300, Taiwan; mjchen@mail.mcu.edu.tw; 7 Department of Mathematics and Statistics and Department of Computer Science, Georgetown University, Washington, DC 20057, USA; chang@georgetown.edu; 8 Department of Ophthalmology, Tri-Service General Hospital, Taipei 114202, Taiwan; 9 Department of Ophthalmology, College of Medicine, National Defense Medical University, Taipei 114201, Taiwan; 10 School of Pharmacy, Kaohsiung Medical University, Kaohsiung 80756, Taiwan

**Keywords:** cynarin, age-related macular degeneration, retinal pigment epithelium, oxidative stress, inflammation, MAPK, NF-κB

## Abstract

**Background:** Age-related macular degeneration (AMD) is a leading cause of irreversible vision loss and is strongly driven by oxidative stress and inflammation. This study investigated the protective effects of cynarin against sodium iodate (NaIO_3_)-induced retinal pigment epithelium (RPE) injury, focusing on the MAPK and NF-κB signaling pathways. **Materials and Methods:** Human ARPE-19 cells were exposed to NaIO_3_, and cell viability was assessed by the MTT assay. Protein expression of MAPK components (p38, JNK, ERK) and the NF-κB pathway was analyzed by Western blotting, and pro-inflammatory cytokine (IL-1β, IL-6, TNF-α) mRNA expression was measured by RT-qPCR. In vivo, NaIO_3_-induced retinal degeneration in C57BL/6 mice was treated with cynarin (3 or 10 mg/kg) for seven days, and retinal changes were evaluated by fundus photography, fluorescein angiography, and OCT. **Results:** Cynarin preserved ARPE-19 cell viability without cytotoxicity. It significantly attenuated NaIO_3_-induced p38 and JNK phosphorylation, IκB degradation, and NF-κB activation while downregulating IL-1β, IL-6, and TNF-α expression. In vivo, cynarin reduced drusen-like lesions, hyperfluorescent abnormalities, and retinal thinning, and dose-dependently suppressed ocular pro-inflammatory cytokines. **Conclusions:** Cynarin protects against oxidative stress-induced retinal degeneration by suppressing MAPK and NF-κB inflammatory signaling, representing a promising therapeutic candidate for preventing or delaying NaIO_3_-induced dry AMD-like retinal injury.

## 1. Introduction

Age-related macular degeneration (AMD) is a leading cause of irreversible vision loss in the elderly population worldwide [1]. The disease primarily affects the macula, resulting in progressive central vision impairment that significantly compromises daily visual function, including reading, facial recognition, and driving. With global population aging, the prevalence of AMD is expected to rise substantially, representing a growing public health and socioeconomic burden [2,3]. AMD is a multifactorial disease driven by aging, environmental exposure, genetic susceptibility, and metabolic stressors. Among these, oxidative stress is considered a central pathogenic mechanism [4,5]. Due to its high oxygen consumption, continuous light exposure, and lipid-rich composition, the retina is particularly vulnerable to oxidative injury. Excessive accumulation of reactive oxygen species (ROS) disrupts redox homeostasis and contributes to retinal pigment epithelium (RPE) dysfunction, inflammation, apoptosis, and photoreceptor degeneration [6,7,8,9]. The RPE plays a critical role in maintaining retinal integrity and outer retinal homeostasis. RPE dysfunction is a key early event in AMD pathogenesis and contributes to drusen formation, chronic inflammation, and progressive neuroretinal degeneration [10]. Mechanistically, oxidative stress activates multiple intracellular signaling cascades, including the mitogen-activated protein kinase (MAPK) and nuclear factor-kappa B (NF-κB) pathways, which promote inflammatory cytokine expression and pro-angiogenic signaling, thereby amplifying retinal injury [11,12,13].

Clinically, AMD is classified into two major subtypes: dry (non-neovascular or atrophic) and wet (neovascular or exudative) forms, which differ markedly in disease mechanisms and therapeutic response [14]. Dry AMD, the predominant form, is characterized by extracellular drusen deposition, progressive RPE atrophy, and photoreceptor loss, ultimately leading to geographic atrophy (GA) and irreversible central vision loss [15,16]. In contrast, wet AMD is defined by choroidal neovascularization, in which abnormal vessels originating from the choroid invade the subretinal or sub-RPE space, resulting in vascular leakage, hemorrhage, and rapid visual deterioration [17]. Currently, anti-vascular endothelial growth factor therapy has significantly improved outcomes in wet AMD. However, treatment options for dry AMD remain limited, with only two drugs targeting the complement system: Pegcetacoplan, an inhibitor of complement component C3, and Avacincaptad pegol, which targets complement component C5. While both agents aim to slow the progression of geographic atrophy in affected patients, they have yet to demonstrate any meaningful improvement in visual outcomes [18]. This unmet clinical need underscores the importance of identifying novel molecular targets and therapeutic strategies for retinal protection in dry AMD [19,20].

Cynarin, a naturally occurring polyphenolic compound isolated from artichoke (*Cynara scolymus*), possesses antioxidant, anti-inflammatory, and cytoprotective properties. It has been shown to modulate oxidative stress-related signaling pathways and reduce ROS accumulation in non-ocular disease models [21,22]. However, its potential role in retinal oxidative injury and AMD-related pathology remains largely unexplored. NaIO_3_ is a well-established oxidizing agent that selectively induces RPE degeneration and is widely used to model dry AMD-like retinal injury [23,24]. NaIO_3_-mediated RPE damage leads to secondary photoreceptor loss and structural alterations resembling human GA. In this study, we investigated the protective effects of cynarin against NaIO_3_-induced retinal degeneration in vivo and oxidative stress-induced injury in human RPE cells in vitro. We further explored the underlying mechanisms with a focus on ROS-associated MAPK and NF-κB signaling pathways and downstream inflammatory responses.

## 2. Materials and Methods

### 2.1. Cynarin

Cynarin was purchased from MedChemExpress (Monmouth Junction, NJ, USA).

### 2.2. Cell Culture

An immortalized human retinal RPE cell line, ARPE-19, was utilized. These cells were obtained from the Biological Resource Collection and Research Center (BCRC, Hsinchu City, Taiwan) at the Food Industry Research and Development Institute, under the catalog number BCRC60383. Being adherent in nature, the cells were cultured in a humidified incubator maintained at 37 °C with 5% CO_2_. The culture medium used was Dulbecco’s Modified Eagle Medium, containing 1% antibiotic–antimycotic solution and 10% fetal bovine serum. Subculturing was performed once the cells reached a confluency level of 80–90%.

### 2.3. Cell Viability Assay

Cell viability was evaluated using the MTT assay. ARPE-19 cells were seeded into 24-well plates and treated with various concentrations of cynarin and NaIO_3_. Following treatment, cells were incubated with MTT solution [3-(4,5-dimethylthiazol-2yl)-2,5-diphenyl-2H-tetrazolium bromide]. The plate was then incubated at 37 °C for approximately 2 h. Following incubation, the medium was discarded, and the resulting formazan crystals were solubilized using dimethyl sulfoxide. After thorough mixing, cell viability was assessed by measuring absorbance at 550 nm using an enzyme-linked immunosorbent assay reader.

### 2.4. Western Blot Analysis

To investigate intracellular protein expression, Western blot analysis was performed. ARPE-19 cells were collected by scraping, lysed by sonication, and centrifuged at 13,200 rpm for 10 min at 4 °C. Protein concentrations in the supernatants were determined using a Pierce protein assay kit (Pierce, Rockford, IL, USA). Equal amounts of protein were separated on 10% SDS-polyacrylamide gels and transferred onto PVDF membranes. Membranes were blocked with 5% skim milk in TBS-T for 1 h and incubated overnight at 4 °C with primary antibodies (1:1000 dilution), followed by incubation with secondary antibodies (1:1000 dilution) for 1 h at room temperature. Protein bands were visualized using a chemiluminescence imaging system (BIOSTEP Celvin^®^, biostep, Burkhardtsdorf, Germany). Original Western blot images are provided in the Supplementary Materials.

### 2.5. Real-Time Quantitative Polymerase Chain Reaction (RT-qPCR) Analysis

Total RNA was extracted from ARPE-19 cells and the homogenized ocular tissues of C57BL/6 mice using TRIzol reagent containing Buffer RL, followed by further purification with an RNA Micro Kit (GeneDireX^®^, Vegas, NV, USA) according to the manufacturer’s instructions. Complementary DNA (cDNA) was synthesized from messenger RNA (mRNA) using the iScript cDNA Synthesis Kit (BIO-RAD, Hercules, CA, USA) via reverse transcription. RT-qPCR was subsequently performed using SYBR Green Master Mix (Applied Biosystems™, Waltham, MA, USA) on a StepOnePlus™ Real-Time PCR System (Thermo Fisher Scientific, Waltham, MA, USA). Briefly, each reaction mixture contained 2 μL of cDNA sample and 8 μL of primer mixture, followed by the addition of 10 μL SYBR Green reagent under light-protected conditions. The sequences of the forward and reverse primers used for each target gene are listed in Table 1.

### 2.6. Animal Experiments

All animal experiments were performed in accordance with the Association for Research in Vision and Ophthalmology (ARVO) Statement for the Use of Animals in Ophthalmic and Vision Research. Male C57BL/6 mice aged four to five weeks were sourced from the National Biotechnology Research Park in Taipei, Taiwan. All of the animal housing and experimental procedures adhered to the guidelines established by the Fu Jen University Laboratory Animal Center. The mice were randomly divided into four groups: (1) control group, which received neither NaIO_3_ nor cynarin, (2) induced group, which received NaIO_3_ but no cynarin, (3) low-dose cynarin group, which received NaIO_3_ and cynarin 3 mg/kg, and (4) high-dose cynarin group, which received NaIO_3_ and cynarin 10 mg/kg.

For the induction of retinal degeneration, NaIO_3_ (40 mg/kg) dissolved in phosphate-buffered saline (PBS) was administered via tail vein injection on the first day of the experiment to mice in Groups 2, 3, and 4, whereas an equivalent volume of PBS was administered to Group 1. Cynarin was subsequently administered intraperitoneally at doses of 3 mg/kg and 10 mg/kg to Groups 3 and 4, respectively, once daily for seven consecutive days beginning on the first experimental day. Groups 1 and 2 received equivalent volumes of PBS during the same treatment period.

### 2.7. Retinal Imaging and Histological Evaluation

Retinal imaging, including CFP, FA, and OCT, was performed on experiment day eight to evaluate retinal morphology and vascular alterations. All imaging procedures were conducted under general anesthesia induced by intraperitoneal injection of a 3:2 mixture of Zoletil™ 50 (zolazepam/tiletamine) and Rompun 20 (xylazine) at a dose of 1 μL/g body weight. Pupil dilation was achieved using 0.5% phenylephrine hydrochloride and 0.5% tropicamide (Santen Pharmaceutical Co., Ltd., Tokyo, Japan). Before image acquisition, each mouse was positioned laterally in front of the imaging device, and the ocular surface was coated with 2% Methocel gel (OmniVision, SA, Neuhausen, Switzerland) to maintain corneal hydration and optical clarity.

OCT images were obtained using the iVue 80 system (Optovue Inc., Fremont, CA, USA), and retinal thickness was quantified after image acquisition using the built-in scale bar integrated into the imaging software. To reduce the potential influence of local measurement variability, we re-analyzed retinal thickness using multiple predefined locations within each OCT scan. The measurements obtained from these predefined locations were averaged to generate one representative retinal-thickness value for each eye.

CFP and FA images were acquired using the Micron IV retinal imaging microscope (Phoenix Research Laboratories, Pleasanton, CA, USA). For the FA examination, 0.05 mL of 10% fluorescein solution was administered via intraperitoneal injection, followed by serial image and video acquisition.

### 2.8. Statistical Analysis

SigmaPlot (Version 15.0, Systat Software, Inc., San Jose, CA, USA) was used for data analysis and graphical presentation. All data are expressed as the mean ± standard error of the mean (SEM). Statistical analyses were performed using Student’s *t*-test or one-way analysis of variance, followed by appropriate post hoc tests where applicable. A *p*-value < 0.05 was considered statistically significant.

## 3. Results

### 3.1. Viability Tests

#### 3.1.1. Cytotoxicity of Cynarin on ARPE-19 Cells

**Figure 1:**
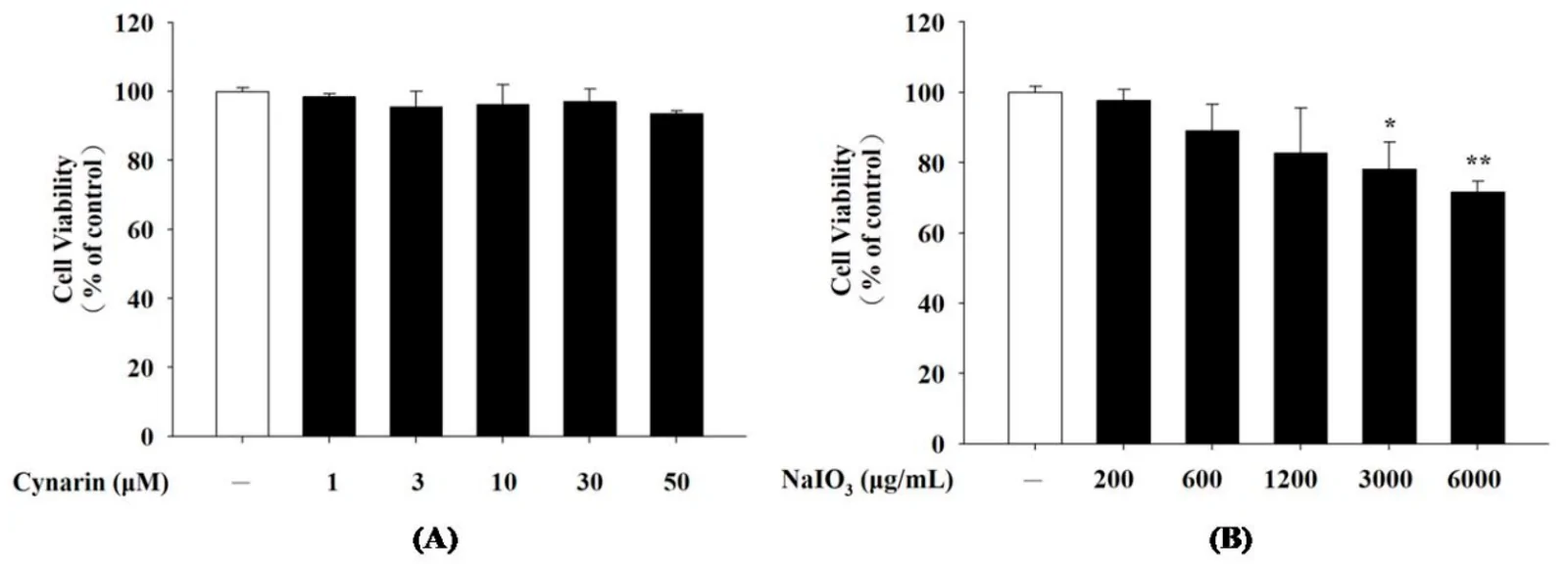
Cell viability of ARPE-19 cells assessed by MTT assay. (**A**) ARPE-19 cells were pretreated with various concentrations of cynarin (0, 1, 3, 10, 30, and 50 μM), and cell viability was subsequently evaluated using the MTT assay. (**B**) ARPE-19 cells were exposed to different concentrations of NaIO_3_ (0, 200, 600, 1200, 3000, and 6000 μg/mL) for 3 h prior to MTT assay analysis. Data are presented as the mean ± SEM from three independent experiments. * *p* < 0.05 and ** *p* < 0.01 versus the control group.

The cytotoxicity of cynarin on ARPE-19 cells was evaluated using the MTT assay. Cells were pre-treated with various concentrations of cynarin (1 µM, 3 µM, 10 µM, 30 µM, and 50 µM) for 24 h. The results demonstrated that cynarin at concentrations ranging from 1 µM to 50 µM did not induce significant cytotoxicity in ARPE-19 cells, maintaining cell viability above 90% across all tested concentrations (Figure 1A). Based on these findings, concentrations of 1 µM, 3 µM, and 10 µM were selected for subsequent experiments.

#### 3.1.2. Effect of NaIO_3_ on ARPE-19 Cell Viability

To determine the optimal concentration of sodium iodate (NaIO_3_) for inducing ARPE-19 cell injury, cells were treated with five different concentrations of NaIO_3_ (200, 600, 1200, 3000, and 6000 μg/mL). As shown in Figure 1B, the results demonstrated that treatment with the lower concentrations of 200 and 600 μg/mL produced minimal effects on cell viability compared with the control group, indicating insufficient induction of pathological injury. In contrast, exposure to 1200 μg/mL NaIO_3_ resulted in a significant reduction in cell viability. Further increases in NaIO_3_ concentration to 3000 and 6000 μg/mL caused marked cytotoxicity, with cell viability decreasing to below 80%, suggesting that NaIO_3_ effectively induced severe cellular damage under these conditions. These findings are consistent with commonly used experimental conditions in AMD-related studies. Therefore, a concentration of 3000 μg/mL NaIO_3_ was selected for subsequent in vitro experiments.

**Figure 2:**
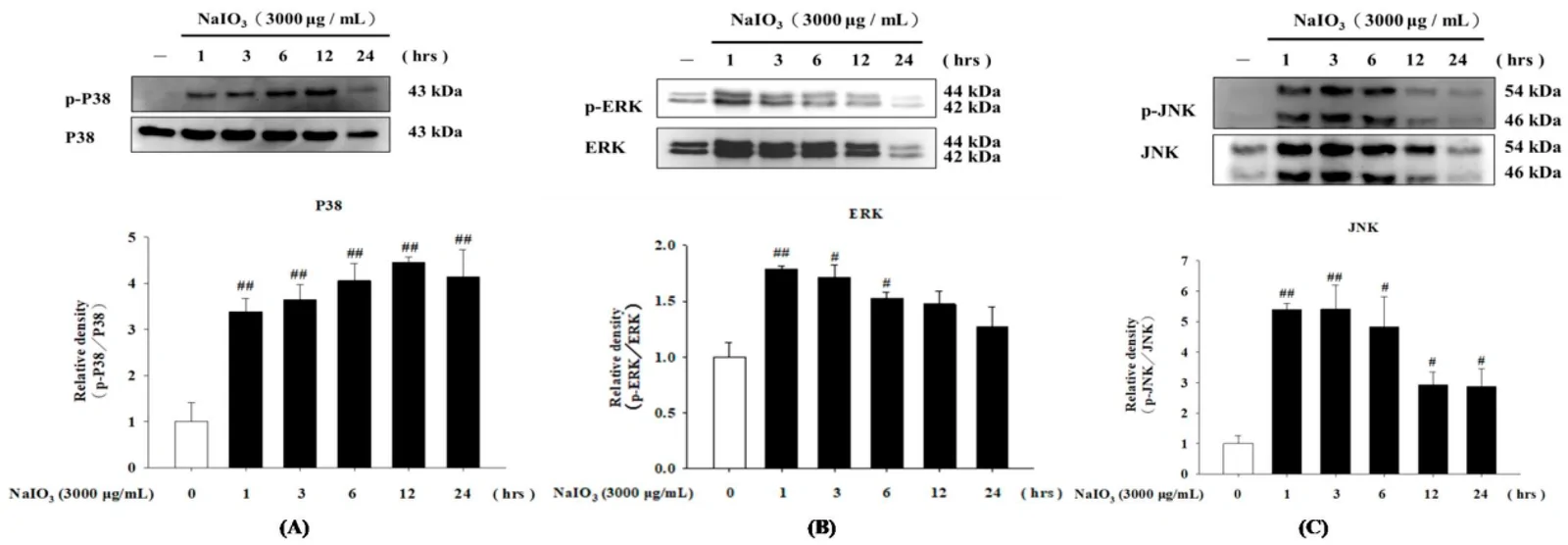
Time-course analysis of NaIO_3_-induced phosphorylation of p38 (**A**), ERK (**B**), and JNK (**C**) in ARPE-19 cells as determined by Western blot analysis. Quantitative data are presented as the mean ± SEM from three independent experiments (*n* = 3). # *p* < 0.05 and ## *p* < 0.01 versus the control group.

Subsequently, to determine the optimal induction duration, a time-course experiment was performed to evaluate the activation of the three major MAPK signaling proteins, p38, ERK, and JNK. As shown in Figure 2A–C, NaIO_3_ exposure induced distinct temporal activation patterns among these MAPK family members.

The phosphorylation of p38 increased significantly as early as 1 h (3.38 ± 0.28-fold vs. control, *p* < 0.001), remained elevated at 3 h (3.63 ± 0.32-fold, *p* < 0.001), and further increased at 6 h (4.05 ± 0.35-fold, *p* < 0.001) and 12 h (4.45 ± 0.11-fold, *p* < 0.001). Although a slight decrease was observed at 24 h (4.13 ± 0.59-fold, *p* < 0.001), p38 phosphorylation remained markedly higher than that of the control group, indicating sustained activation throughout the experimental period.

In contrast, ERK phosphorylation exhibited a transient activation pattern, increasing significantly at 1 h (1.79 ± 0.02-fold, *p* < 0.01) and 3 h (1.71 ± 0.11-fold, *p* < 0.05), but gradually declining thereafter to 1.52 ± 0.05-fold at 6 h, 1.47 ± 0.11-fold at 12 h, and 1.27 ± 0.18-fold at 24 h, with no significant differences from the control group after 3 h (*p* > 0.05).

Similarly, JNK phosphorylation was rapidly induced, reaching 5.39 ± 0.20-fold at 1 h (*p* < 0.01) and remaining at a comparable level at 3 h (5.40 ± 0.79-fold, *p* < 0.01). Thereafter, JNK phosphorylation gradually decreased to 4.48 ± 0.98-fold at 6 h (*p* < 0.05), 2.92 ± 0.42-fold at 12 h (*p* < 0.05), and 2.28 ± 0.58-fold at 24 h (*p* < 0.05).

These findings indicate that NaIO_3_ induced distinct temporal activation profiles among MAPK family members. ERK and JNK exhibited rapid but transient phosphorylation following oxidative stress, whereas p38 activation was sustained throughout the observation period, suggesting prolonged cellular stress signaling. To capture the robust activation of all three MAPK pathways, particularly the early activation of ERK and JNK while maintaining consistently elevated p38 phosphorylation, an induction duration of 3 h was selected for all subsequent in vitro experiments.

In addition, excessively short induction periods may not fully reflect oxidative stress-mediated signaling activation, whereas prolonged exposure could lead to substantial cytotoxicity and introduce experimental bias. Based on these considerations, 3 h of NaIO_3_ treatment was selected as the optimal induction duration for subsequent in vitro experiments.

### 3.2. Protective Effect of Cynarin Against NaIO_3_-Induced Cytotoxicity

The potential protective effect of cynarin against NaIO_3_-induced cytotoxicity was investigated by pretreating ARPE-19 cells with 3000 μg/mL for 3 h, followed by different concentrations of cynarin (1 µM, 3 µM, and 10 µM).

#### 3.2.1. Cynarin Reduced NaIO_3_-Induced MAPK Phosphorylation

**Figure 3:**
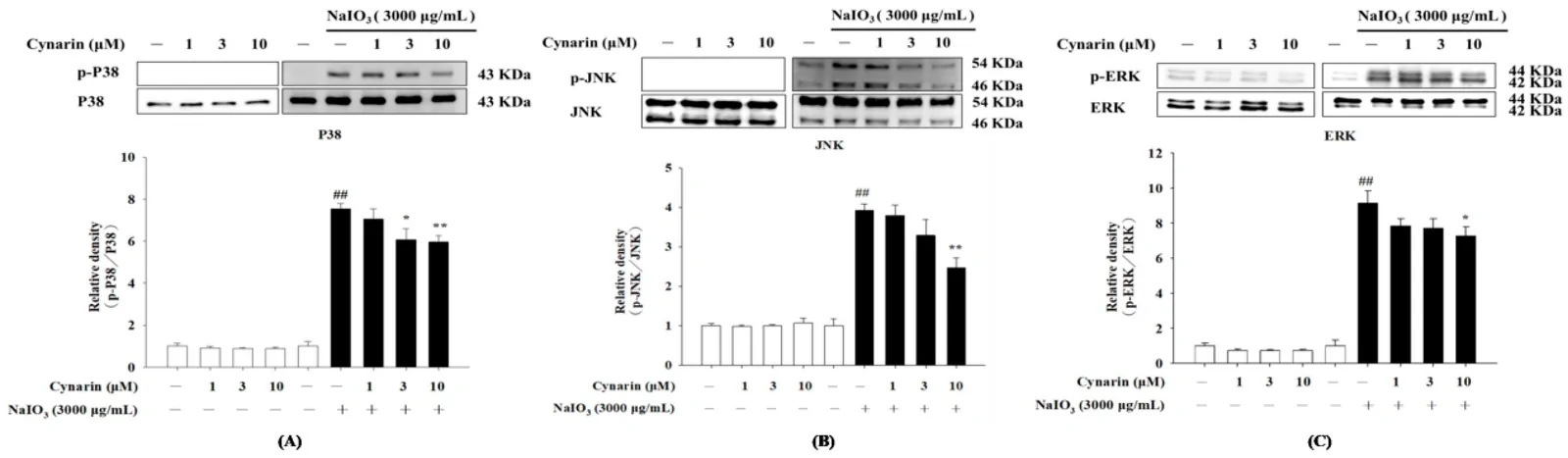
Effects of cynarin on the expression of MAPK signaling pathway-related proteins, including p38 (**A**), JNK (**B**), and ERK (**C**), in NaIO_3_-treated ARPE-19 cells. Protein expression levels were analyzed by Western blotting. Data are presented as the mean ± SEM from five independent experiments (*n* = 5). ## *p* < 0.01 versus the control group; * *p* < 0.05 and ** *p* < 0.01 versus the NaIO_3_-treated group.

The results demonstrated that cynarin exerted a protective effect against NaIO_3_-induced cellular injury, and this protective effect became more pronounced with increasing concentrations of cynarin. Compared with the control group, NaIO_3_ treatment significantly increased the phosphorylation levels of p38, ERK, and JNK to 7.53 ± 0.24-fold, 2.98 ± 0.15-fold, and 11.40 ± 0.98-fold, respectively (all *p* < 0.001). Cynarin treatment reduced p38 phosphorylation to 7.21 ± 0.37-fold, 6.07 ± 0.50-fold, and 5.95 ± 0.30-fold at concentrations of 1, 3, and 10 μM, respectively, with significant reductions observed at 3 μM (*p* = 0.0030) and 10 μM (*p* = 0.0037) compared with the NaIO_3_-treated group (Figure 3A). JNK phosphorylation was reduced to 2.60 ± 0.26-fold, 2.34 ± 0.40-fold, and 1.85 ± 0.24-fold following treatment with 1, 3, and 10 μM cynarin, respectively, with a significant reduction observed only at 10 μM (*p* = 0.0021) (Figure 3B). ERK phosphorylation also decreased to 10.07 ± 0.50-fold, 10.26 ± 0.57-fold, and 9.27 ± 0.63-fold after treatment with 1, 3, and 10 μM cynarin, respectively; however, these reductions did not reach statistical significance (*p* = 0.138, 0.135, and 0.0567, respectively) (Figure 3C). Collectively, these findings indicate that cynarin attenuates NaIO_3_-induced activation of the MAPK signaling pathway in ARPE-19 cells.

#### 3.2.2. Cynarin Reduced NaIO_3_-Induced NF-κB Signaling Pathway

**Figure 4:**
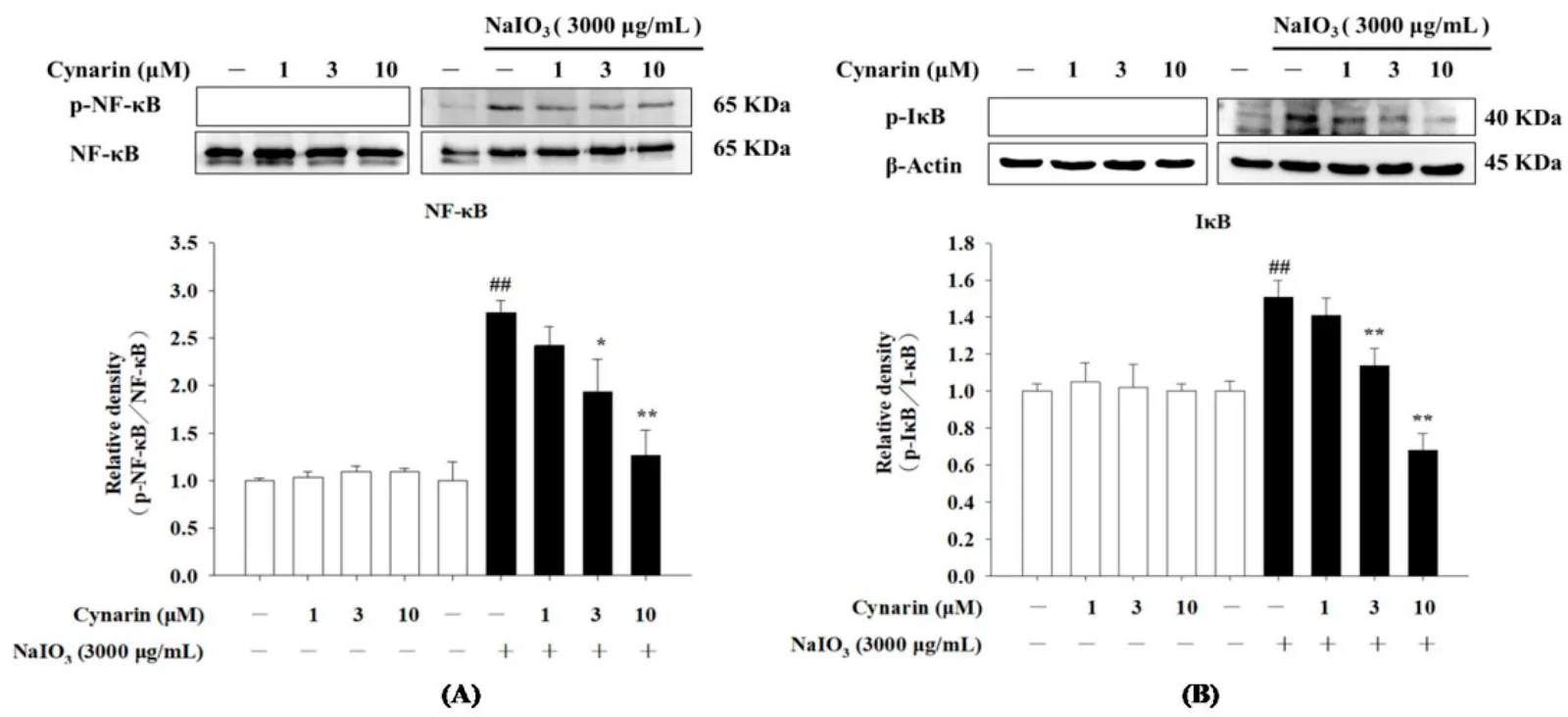
Effects of cynarin on the expression of proteins involved in the NF-κB signaling pathway, including NF-κB (**A**) and IκB (**B**), in NaIO_3_-treated ARPE-19 cells. Protein expression levels were determined by Western blot analysis. Data are presented as the mean ± SEM from five independent experiments (*n* = 5). ## *p* < 0.01 versus the control group; * *p* < 0.05 and ** *p* < 0.01 versus the NaIO_3_-treated group.

The results demonstrated that cynarin attenuated the activation of the NF-κB signaling pathway induced by NaIO_3_. Compared with the control group, NaIO_3_ treatment significantly increased the phosphorylation level of NF-κB to 2.75 ± 0.12-fold (*p* < 0.001). Cynarin treatment reduced NF-κB phosphorylation to 2.41 ± 0.12-fold, 1.93 ± 0.27-fold, and 1.25 ± 0.26-fold at concentrations of 1, 3, and 10 μM, respectively. Although the reduction at 1 μM was not statistically significant (*p* = 0.625), significant decreases were observed at 3 μM (*p* = 0.0168) and 10 μM (*p* = 0.000702) compared with the NaIO_3_-treated group (Figure 4A).

Similarly, NaIO_3_ treatment significantly increased the phosphorylation level of IκB to 1.50 ± 0.09-fold compared with the control group (*p* < 0.001). Cynarin treatment reduced IκB phosphorylation to 1.41 ± 0.09-fold, 1.13 ± 0.09-fold, and 0.67 ± 0.09-fold at concentrations of 1, 3, and 10 μM, respectively. The reduction was not statistically significant at 1 μM (*p* = 0.481), whereas significant decreases were observed at 3 μM (*p* = 0.0239) and 10 μM (*p* = 0.000228) compared with the NaIO_3_-treated group (Figure 4B). Collectively, these findings suggest that the protective effect of cynarin against NaIO_3_-induced oxidative injury is associated with suppression of the NF-κB signaling pathway.

#### 3.2.3. Cynarin Downregulated Pro-Inflammatory Cytokine mRNA Expression Induced by NaIO_3_

**Figure 5:**
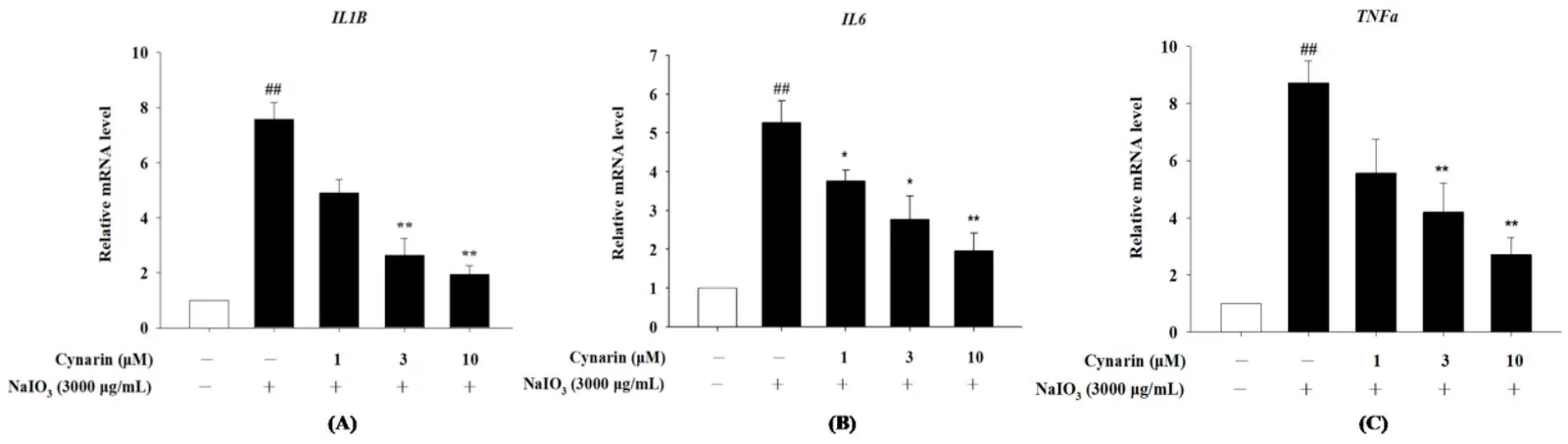
Effects of cynarin on inflammatory cytokine mRNA expression in ARPE-19 cells. RT-qPCR analysis was performed to determine the relative mRNA expression levels of (**A**) IL-1β, (**B**) IL-6, and (**C**) TNF-α following cynarin treatment in NaIO_3_-stimulated ARPE-19 cells. Data are presented as the mean ± SEM from five independent experiments (*n* = 5). ## *p* < 0.01 versus the control group; * *p* < 0.05 and ** *p* < 0.01 versus the NaIO_3_-treated group.

The mRNA expression levels of the pro-inflammatory cytokines, including interleukin-1β (IL-1β), interleukin-6 (IL-6), and tumor necrosis factor-α (TNF-α), were analyzed by RT-qPCR following NaIO_3_ exposure. The results demonstrated that NaIO_3_ treatment markedly increased the mRNA expression of IL-1β, IL-6, and TNF-α compared with the control group (Figure 5A–C).

Specifically, NaIO_3_ treatment significantly increased IL-1β mRNA expression to 7.57 ± 0.61-fold compared with the control group (*p* < 0.001). Cynarin treatment reduced IL-1β expression to 4.91 ± 0.48-fold, 2.26 ± 0.60-fold, and 1.93 ± 0.33-fold at concentrations of 1, 3, and 10 μM, respectively. All three concentrations significantly decreased IL-1β expression compared with the NaIO_3_-treated group (*p* = 0.00922, *p* = 0.000439, and *p* = 0.0000397, respectively) (Figure 5A).

Similarly, NaIO_3_ treatment significantly increased IL-6 mRNA expression to 5.25 ± 0.55-fold compared with the control group (*p* < 0.001). Cynarin treatment reduced IL-6 expression to 3.76 ± 0.27-fold, 0.77 ± 0.60-fold, and 1.96 ± 0.45-fold at concentrations of 1, 3, and 10 μM, respectively. Significant reductions were observed at all concentrations compared with the NaIO_3_-treated group (Figure 5B).

Likewise, NaIO_3_ treatment significantly increased TNF-α mRNA expression to 8.71 ± 0.78-fold compared with the control group (*p* < 0.001). Cynarin treatment reduced TNF-α expression to 5.56 ± 1.18-fold, 4.20 ± 1.00-fold, and 2.70 ± 0.59-fold at concentrations of 1, 3, and 10 μM, respectively. Although the reduction at 1 μM was not statistically significant, significant decreases were observed at 3 μM and 10 μM compared with the NaIO_3_-treated group (Figure 5C).

Overall, these findings indicate that cynarin suppresses NaIO_3_-induced inflammatory responses by downregulating the expression of pro-inflammatory cytokines in a concentration-dependent manner.

### 3.3. Protective Effect of Cynarin Against NaIO_3_-Induced Retinal Degeneration in Mice

**Figure 6:**
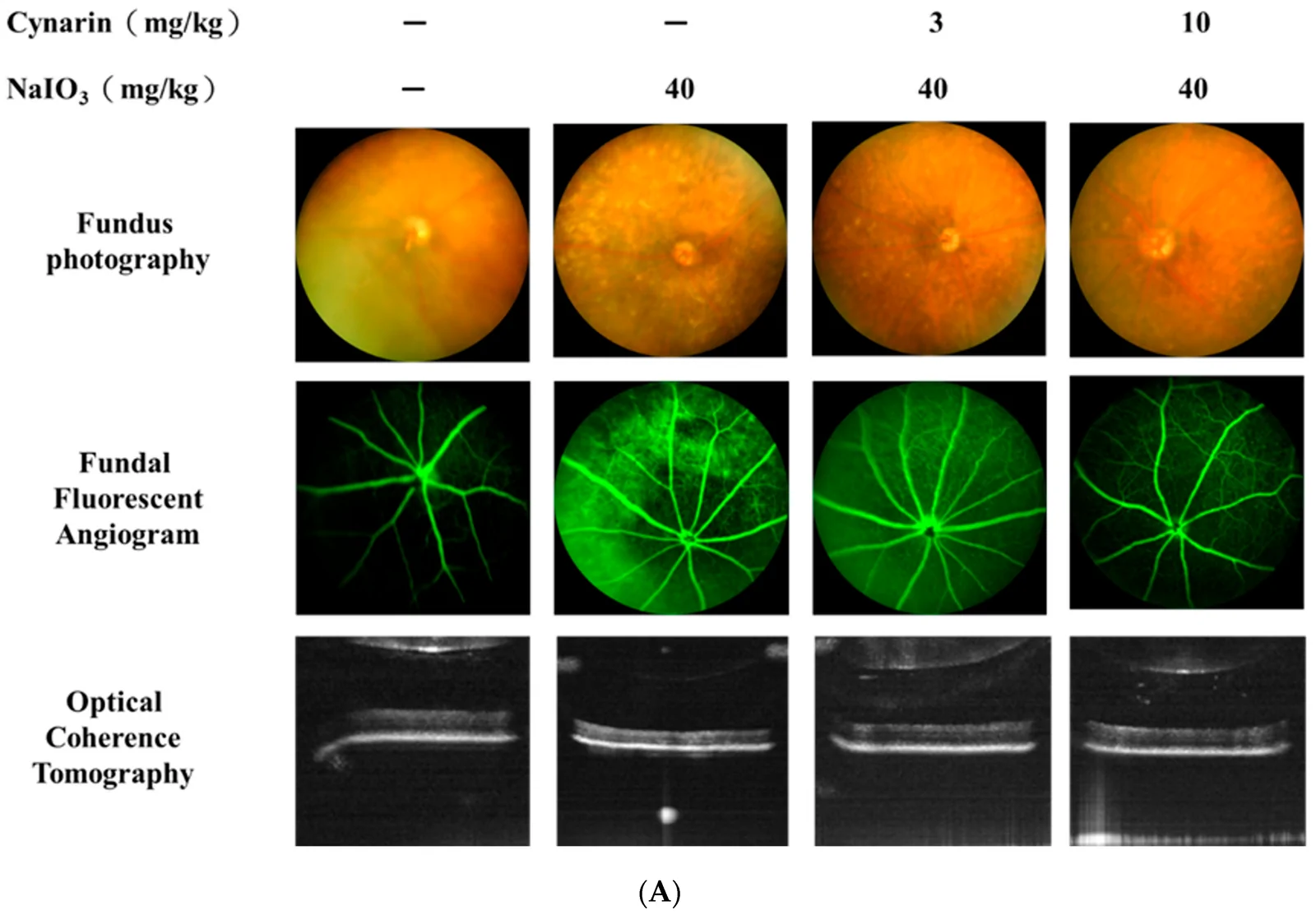

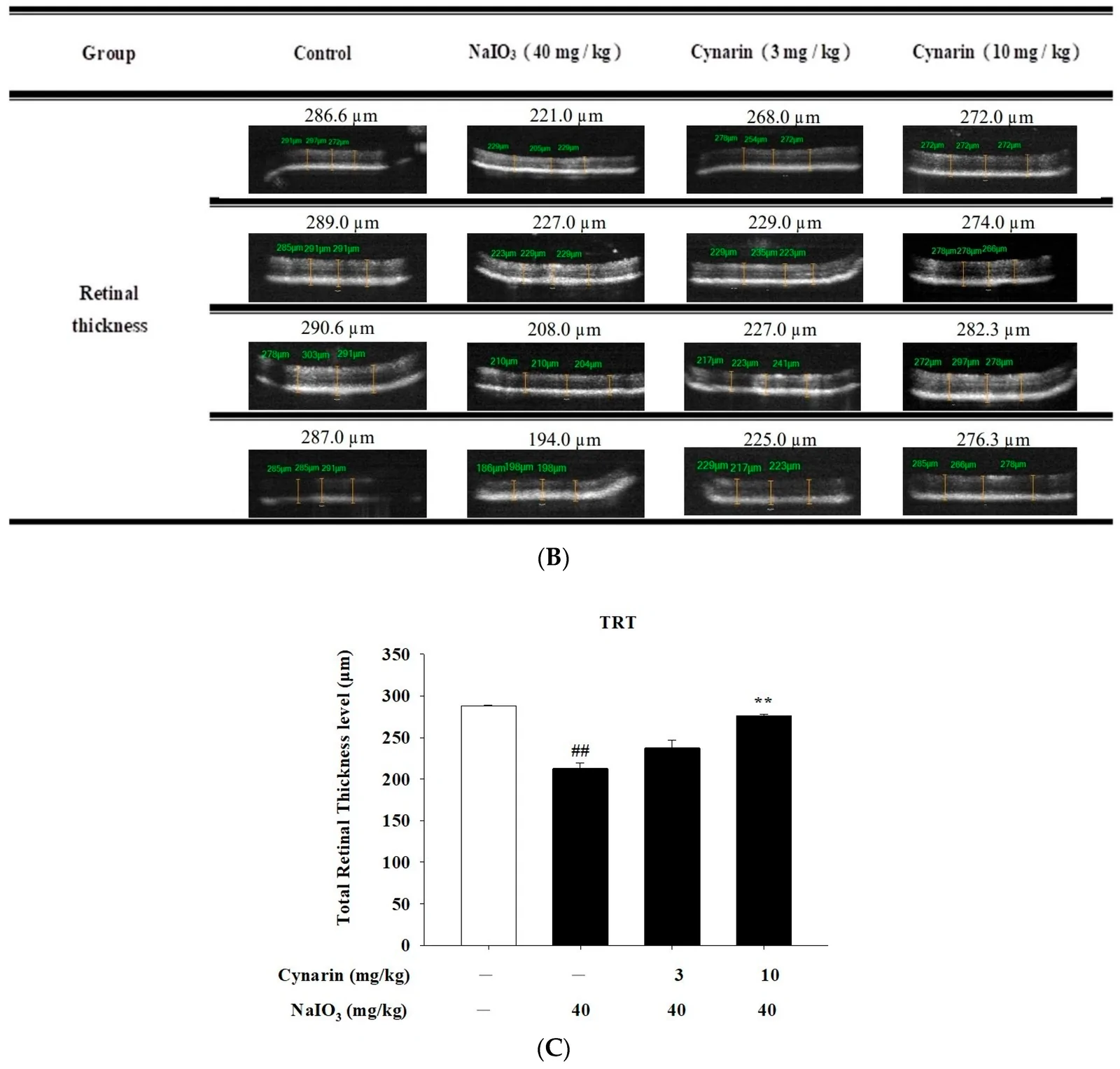
(**A**) Representative color fundus photography (CFP), fluorescein angiography (FA), and optical coherence tomography (OCT) images of C57BL/6 mice from each experimental group. The yellowish drusen-like deposits observed on CFPs following NaIO_3_ treatment were significantly reduced after administration of cynarin. Correspondingly, multiple round hyperfluorescent lesions identified on FA emerged after NaIO_3_ treatment and showed a marked decrease following cynarin treatment. (**B**) Quantitative analysis of retinal thickness measured by OCT in each group. (**C**) Effects of cynarin treatment on retinal thickness in NaIO_3_-induced retinal injury in C57BL/6 mice. Data are presented as the mean ± SEM (*n* = 4). ## *p* < 0.01 versus the control group; ** *p* < 0.01 versus the NaIO_3_-treated group.

The results demonstrated that cynarin exerted a protective effect against NaIO_3_-induced retinal injury in vivo. As shown in Figure 6A, no apparent abnormalities were observed in either color fundus photography (CFP) or fluorescein angiography (FA) in the PBS-treated control group. In contrast, mice treated with NaIO_3_ and PBS exhibited numerous yellowish drusen-like deposits on CFP images. Correspondingly, FA revealed multiple round hyperfluorescent lesions with poorly defined borders, which were likely attributable to a window-defect associated with drusen-like lesion formation. Optical coherence tomography (OCT) further demonstrated marked retinal thinning in the NaIO_3_-treated group.

Daily administration of cynarin markedly alleviated these pathological changes. Compared with the NaIO_3_-treated group, both the 3 mg/kg and 10 mg/kg cynarin-treated groups exhibited fewer drusen-like lesions and less retinal thinning. Quantitative analysis demonstrated that NaIO_3_ treatment significantly reduced retinal thickness to 216.00 ± 7.62 μm compared with the control group (*p* < 0.001). Cynarin treatment significantly increased retinal thickness to 233.75 ± 7.31 μm in the 3 mg/kg group (*p* = 0.002 vs. NaIO_3_) and to 278.25 ± 6.71 μm in the 10 mg/kg group (*p* = 0.002 vs. NaIO_3_). Collectively, these findings demonstrate that cynarin effectively attenuated NaIO_3_-induced retinal degeneration in vivo. Representative OCT images and quantitative analyses of retinal thickness are presented in Figure 6B and Figure 6C, respectively.

### 3.4. Quantitative RT–PCR Analysis of Inflammatory Cytokine Expression

**Figure 7:**
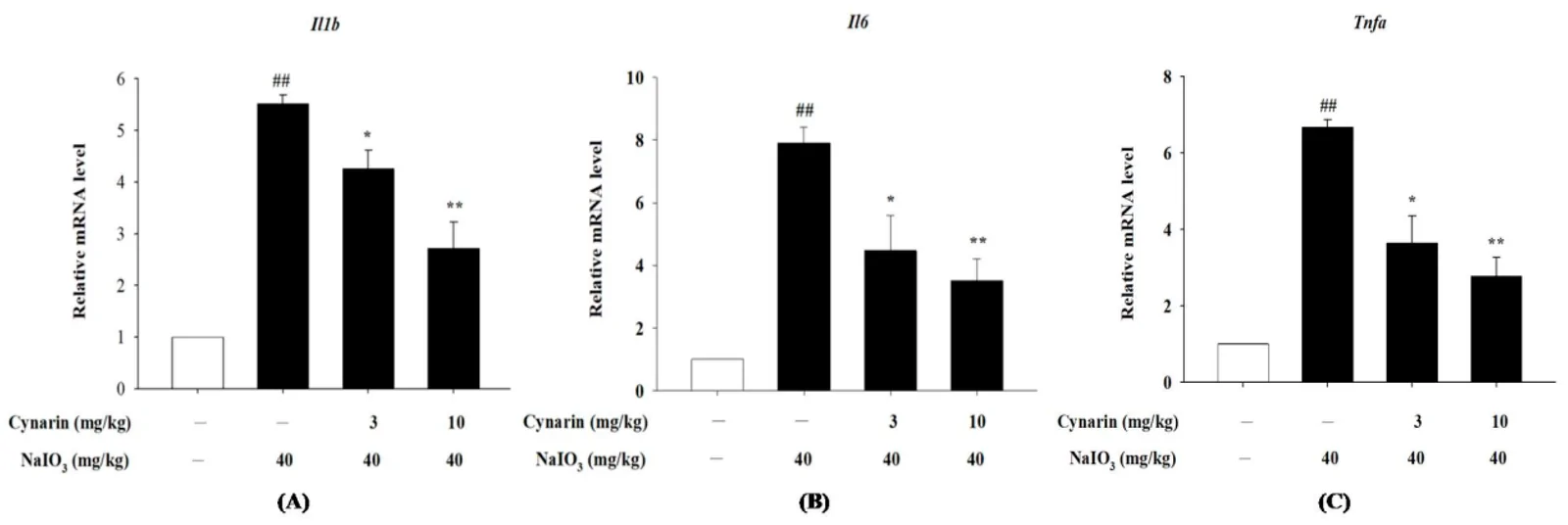
Effects of cynarin on inflammatory cytokine mRNA expression in eyeball tissues of NaIO_3_-induced C57BL/6 mice. RT-qPCR analysis was performed to quantify the relative mRNA expression levels of (**A**) IL-1β, (**B**) IL-6, and (**C**) TNF-α in eyeball tissues following NaIO_3_ induction and cynarin treatment. Data are presented as the mean ± SEM from four independent experiments (*n* = 4). ## *p* < 0.01 versus the control group; * *p* < 0.05 and ** *p* < 0.01 versus the NaIO_3_-treated group.

The results demonstrated that cynarin attenuated NaIO_3_-induced inflammatory responses in vivo. After imaging analysis, the mice were sacrificed, and both eyeballs were collected for RT-qPCR analysis following whole-eye homogenization. As shown in Figure 7A–C, NaIO_3_ treatment significantly increased the mRNA expression levels of the pro-inflammatory cytokines IL-1β, IL-6, and TNF-α compared with the control group. Specifically, IL-1β expression increased to 5.52 ± 0.16-fold (*p* < 0.001), IL-6 increased to 7.57 ± 0.49-fold (*p* < 0.001), and TNF-α increased to 6.75 ± 0.22-fold (*p* < 0.001).

Daily administration of cynarin significantly attenuated these NaIO_3_-induced increases in a dose-dependent manner. Compared with the NaIO_3_-treated group, treatment with 3 mg/kg cynarin reduced IL-1β mRNA expression to 4.26 ± 0.35-fold (*p* = 0.0121), while 10 mg/kg cynarin further decreased expression to 2.71 ± 0.51-fold (*p* = 0.00589). Likewise, IL-6 mRNA expression was significantly reduced to 5.29 ± 0.98-fold (*p* = 0.00488) and 3.93 ± 0.70-fold (*p* = 0.00602) following treatment with 3 and 10 mg/kg cynarin, respectively. TNF-α mRNA expression was also significantly decreased to 3.58 ± 0.91-fold (*p* = 0.0150) and 2.50 ± 0.52-fold (*p* = 0.000319) in the 3 and 10 mg/kg cynarin-treated groups, respectively.

These findings are consistent with the in vitro results and further demonstrate that cynarin effectively suppresses NaIO_3_-induced inflammatory cytokine expression in vivo in a dose-dependent manner.

## 4. Discussion

AMD, particularly the dry form, is a multifactorial retinal degenerative disease and a leading cause of irreversible vision loss in the aging population. Oxidative stress and chronic inflammation have been strongly implicated in dry AMD pathogenesis [25,26]. These factors promote excessive production of ROS, leading to oxidative damage, mitochondrial dysfunction, and RPE cell injury, which are key events in the initiation and progression of dry AMD [26,27]. In addition to oxidative stress, sustained inflammatory responses further exacerbate retinal degeneration [28,29]. Activation of innate immune signaling in RPE cells and the retinal microenvironment induces the production of pro-inflammatory cytokines and chemokines, contributing to a chronic inflammatory state [30]. Importantly, oxidative stress and inflammation are tightly interconnected, as ROS can activate redox-sensitive signaling pathways such as NF-κB and MAPK, thereby amplifying inflammatory cascades and accelerating RPE dysfunction [31,32,33]. Cynarin has been widely reported to possess potent antioxidant and anti-inflammatory activities [34]. Previous studies have demonstrated that cynarin can effectively scavenge ROS, reduce lipid peroxidation, and enhance cellular antioxidant defense mechanisms [35]. This study systematically evaluated cynarin’s protective effects on ARPE-19 cells under NaIO_3_-induced stress, offering insights into its mechanisms and potential application for dry AMD. In the present study, cynarin exhibited no significant cytotoxicity in ARPE-19 cells at concentrations up to 50 μM, with MTT assay results demonstrating cell viability exceeding 90%. These findings indicate that cynarin did not adversely affect ARPE-19 cell survival under the tested conditions, thereby confirming its suitability and safety for subsequent in vitro experiments. This observation is consistent with previous studies reporting that cynarin possesses potent antioxidant and anti-inflammatory bioactivities while exhibiting minimal cytotoxic effects in various cellular models [33]. The favorable safety profile of cynarin further supports its potential application as a therapeutic candidate for oxidative stress-related retinal diseases.

The RPE plays a pivotal role in maintaining retinal homeostasis and photoreceptor survival through the regulation of nutrient transport, phagocytosis of photoreceptor outer segments, maintenance of the outer blood–retinal barrier, immune modulation, and protection against oxidative injury [36]. However, under pathological conditions, prolonged oxidative stress and chronic inflammation can disrupt RPE function and contribute to the development and progression of AMD [28,37]. In dry AMD, due to its high oxygen consumption, continuous exposure to light, and abundance of polyunsaturated fatty acids, the retina is particularly susceptible to oxidative damage [38,39,40]. Excessive accumulation of ROS induces mitochondrial dysfunction, lipid peroxidation, DNA damage, and cellular senescence in RPE cells, ultimately leading to photoreceptor degeneration and retinal atrophy [37,39,40].

In addition to oxidative injury, persistent inflammatory responses further exacerbate retinal degeneration. Oxidative stress stimulates the activation of redox-sensitive intracellular signaling pathways, particularly NF-κB and MAPK signaling cascades [32,33,41,42], which are recognized as central mediators in AMD pathogenesis. Activation of the MAPK pathway, including p38, JNK, and ERK, promotes apoptotic signaling, inflammatory cytokine production, and stress-induced cellular dysfunction in RPE cells [43,44]. Concurrently, activation of NF-κB signaling induces the transcription of multiple pro-inflammatory cytokines and chemokines, including IL-1β, IL-6, and TNF-α, thereby amplifying chronic inflammation, immune cell recruitment, and retinal injury [45,46]. Furthermore, increasing evidence suggests that oxidative stress and inflammation form a self-perpetuating cycle in AMD pathogenesis. ROS-mediated activation of NF-κB and MAPK pathways enhances inflammatory signaling, while inflammatory mediators further increase oxidative stress and mitochondrial dysfunction, accelerating RPE cell death and retinal degeneration [28,47,48,49]. Sustained activation of NF-κB and MAPK signaling pathways contributes to progressive RPE degeneration, drusen formation, progression to dry AMD, and eventual GA. GA, the advanced form of dry AMD, is characterized by progressive degeneration of the RPE, photoreceptor loss, and choriocapillaris atrophy, ultimately leading to irreversible central vision loss [50,51,52]. Persistent oxidative stress promotes the accumulation of lipofuscin and drusen components, which further exacerbate retinal injury and inflammatory activation in GA lesions [50,53]. Sustained NF-κB activation also contributes to complement dysregulation, immune cell recruitment, and chronic para-inflammation, which are hallmarks of GA pathology [32].

Based on the above, oxidative stress and inflammation play important roles in the pathogenesis of dry AMD and its progression to the end stage. Therefore, therapeutic strategies targeting oxidative stress-associated NF-κB and MAPK activation may provide potential benefits in preventing the development of dry AMD and slowing its progression to GA. In this study, ARPE-19 cells exposed to NaIO_3_ exhibited a dose-dependent reduction in viability. Cynarin treatment enhanced ARPE-19 cell viability in a concentration-dependent manner. This protective effect arises from cynarin’s ability to significantly reduce NaIO_3_-induced inflammatory retinal injury. Among the downstream regulatory proteins of the MAPK family, ERK signaling is primarily associated with cell proliferation, differentiation, and survival, whereas p38 and JNK are more closely involved in apoptosis and inflammatory responses [54,55]. In the present study, cynarin markedly suppressed the activation of p38 and JNK, suggesting that it effectively attenuated oxidative stress-induced inflammatory signaling. In contrast, the inhibitory effect of cynarin on ERK activation was relatively modest, indicating that cynarin did not substantially interfere with cellular repair and survival signaling pathways. These findings suggest that cynarin exerts a selective cytoprotective effect, primarily through suppression of inflammation-associated apoptotic signaling under oxidative stress conditions. Oxidative stress and external stimuli promote the degradation of IκB, thereby enabling the translocation of the NF-κB complex into the nucleus, where it activates the transcription of multiple genes associated with inflammation, apoptosis, and immune responses [56,57,58]. In the present in vitro study, cynarin was shown to significantly attenuate IκB degradation, thereby reducing the nuclear translocation of NF-κB dimers and subsequently suppressing the expression of a series of pro-inflammatory genes.

Collectively, these findings indicate that the effects of cynarin on the MAPK and NF-κB signaling pathways are closely interconnected, jointly contributing to a multi-layered protective mechanism against oxidative stress-induced retinal injury. These protective effects were further validated in the in vivo experiments. Cynarin effectively ameliorated NaIO_3_-induced drusen-like lesions in C57BL/6 mice and significantly attenuated retinal thinning. Moreover, analysis of retinal tissues demonstrated that these protective effects were accompanied by suppression of pro-inflammatory cytokine gene expression, including IL-1β, IL-6, and TNF-α. These findings suggest that cynarin exerts retinal protective effects primarily through modulation of oxidative stress-associated inflammatory signaling pathways.

Several limitations of the present study should be acknowledged. First, although the NaIO_3_-induced retinal degeneration model is widely used to mimic oxidative stress-mediated RPE injury and dry AMD-like pathology, it does not fully recapitulate the chronic and multifactorial nature of human dry AMD and geographic atrophy. The acute oxidative injury induced by NaIO_3_ may differ from the slowly progressive degenerative processes observed in patients. Second, the in vitro experiments were performed exclusively using the ARPE-19 cell line. Although ARPE-19 cells are commonly used in retinal research, they may not completely reflect the physiological characteristics and functional heterogeneity of native human RPE cells. Nonetheless, ARPE-19 cells provide preliminary screening value for assessing therapeutic candidates and exploring potential treatments for retinal diseases. Future research should validate these findings with primary RPE cells to enhance understanding of therapeutic implications in a more physiologically relevant context. Third, this study primarily focused on inflammatory signaling pathways, including MAPK and NF-κB, without directly quantifying intracellular ROS production, mitochondrial dysfunction, apoptosis-related proteins, or complement pathway activation, all of which are known to contribute to dry AMD pathogenesis. In addition, although cynarin demonstrated protective effects in both in vitro and in vivo models, the precise molecular targets and pharmacokinetic properties of cynarin within retinal tissues remain unclear. Fourth, the relatively short experimental duration limited the evaluation of long-term retinal protection and functional visual outcomes. Further investigations incorporating chronic AMD models, electroretinography, histopathological analyses, and comprehensive mechanistic studies are warranted to better clarify the therapeutic potential of cynarin for dry AMD and geographic atrophy. Fifth, intracellular and tissue ROS levels were not directly measured; therefore, suppression of oxidative stress should be regarded as a plausible upstream mechanism rather than a mechanism directly demonstrated by the present experiments. MAPK and NF-κB signaling was evaluated only in ARPE-19 cells and was not directly confirmed in retinal tissues following cynarin treatment. Sixth, inflammatory cytokine expression in vivo was measured using whole-eye homogenates, preventing attribution of these changes specifically to the retina or RPE. Tissue-resolved analyses of the neural retina and RPE/choroid complex will therefore be required in future studies. For the Western blot analyses, phosphorylation of p38, ERK, JNK, and NF-κB was normalized to the corresponding total proteins, whereas phospho-IκBα was normalized to β-actin. Seventh, cynarin was administered intraperitoneally in the present proof-of-concept study. Although biological activity after oral administration has been reported, the oral pharmacokinetics of cynarin, contribution of circulating metabolites, and retinal exposure remain insufficiently characterized. These issues will require dedicated pharmacokinetic and oral-dose efficacy studies before clinical translation can be considered. Finally, because cynarin was not directly compared with other antioxidants in the present study, no conclusions can be drawn regarding its relative efficacy compared with other antioxidants.

## 5. Conclusions

In conclusion, the present study demonstrated that cynarin exerts significant protective effects against NaIO_3_-induced oxidative retinal injury in both ARPE-19 cells and C57BL/6 mice. Cynarin effectively attenuated oxidative stress-associated inflammatory responses by suppressing activation of the MAPK and NF-κB signaling pathways, leading to reduced expression of pro-inflammatory cytokines, including IL-1β, IL-6, and TNF-α. In vitro, cynarin reduced NaIO_3_-induced cytotoxicity and inhibited phosphorylation of p38 and JNK while exerting a comparatively modest effect on ERK signaling, suggesting a selective anti-inflammatory and cytoprotective mechanism. In vivo, cynarin ameliorated drusen-like retinal lesions and attenuated retinal thinning in NaIO_3_-treated mice, accompanied by downregulation of inflammatory cytokine expression in ocular tissues. Collectively, these findings indicate that cynarin may protect RPE cells and retinal structures from oxidative stress-mediated degeneration through modulation of ROS-associated inflammatory signaling pathways. Given the critical roles of oxidative stress and chronic inflammation in the pathogenesis of dry AMD and geographic atrophy, cynarin may represent a promising therapeutic candidate for preventing or slowing the progression of NaIO_3_-induced dry AMD-like retinal injury. Further studies are warranted to elucidate its long-term efficacy, molecular targets, and translational potential in retinal degenerative diseases.

## Figures and Tables

**Table 1:** Primers of reverse transcription PCR analysis for genes.

Primer	Forward	Reverse
Human GAPDH	5′-CCACCCATGGCAAATTCCATGGCA-3′	5′-TCTAGACGGCAGGTCAGGTCCACC-3′
Human IL-1β	5′-AAACAGATGAACTGCTCCTTCCAGG-3′	5′-ACTTCTTGTGGTGAACAACGAGGT-3′
Human IL-6	5′-CCTTCTCCACAAGCGCCTTC-3′	5′-GGCAAGTCTCCTCATTGAATC-3′
Human TNF–α	5′-CCTCTCTCTAATCAGCCCTCTG-3′	5′-GAGGACCTGGGAGTAGATGAG-3′
Murine GAPDH	5′-CCCGCTTCGCTCTCTGCTCC-3′	5′-ACCAGGCGCCAATACGACC-3′
Murine IL-1β	5′-ACTACAGGCTCCGAGATGAACAAC-3′	5′-CCCAAGGCCACAGGTATTTT-3′
Murine IL-6	5′-GACAAAGCCAGAGTCCTTCAGAGAG-3′	5′-CTAGGTTTGCCGAGTAGATCTC-3′
Murine TNF–α	5′-TGCACCACCATCAAGGACTCAAAT-3′	5′-CCCCGGCCTTCCAAATAAATACAT-3′

## Data Availability

The original contributions presented in this study are included in this article/Supplementary Materials. Further inquiries can be directed to the corresponding authors.

## Supplementary Materials

The following supporting information can be downloaded at: https://www.mdpi.com/article/10.3390/biom16091227/s1; Original Western blot images are provided in the Supplementary Materials.

## Abbreviations

The following abbreviations are used in this manuscript:

RPE	Retinal pigment epithelium
MAPK	Mitogen-activated protein kinase
NF-κB	Nuclear factor-kappa B
JNK	c-Jun N-terminal kinase
ERK	Extracellular signal-regulated kinase
IκB	Inhibitor of κB
RT-qPCR	Quantitative real-time polymerase chain reaction
IL	Interleukin
TNF-α	Tumor necrosis factor-α
CFP	Color fundus photography
FA	Fluorescein angiography
OCT	Optical coherence tomography
AMD	Age-related macular degeneration
ROS	Reactive oxygen species
GA	Geographic atrophy
BCRC	Biological Resource Collection and Research Center
PBS	Phosphate-buffered saline
SEM	Standard error of the mean
MTT	3-(4,5-dimethylthiazol-2yl)-2,5-diphenyl-2H-tetrazolium bromide
