# Supplementary material for: Cynarin Alleviates Sodium Iodate-Induced Retinal Pigment Epithelium Injury by Regulating Oxidative Stress and Inflammation
Source: Biomolecules. 2026 Aug 24;16(9):1227. doi: 10.3390/biom16091227 (PMC13604191; doi:10.3390/biom16091227)

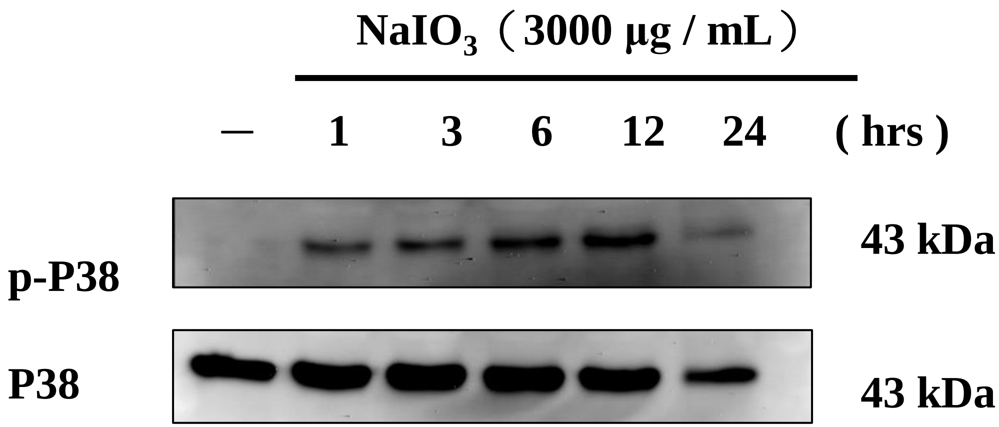
NaIO3（3000 μg / mL）
— 1 3 6 12 24 (hrs)
p-P38
43 kDa
P38
43 kDa

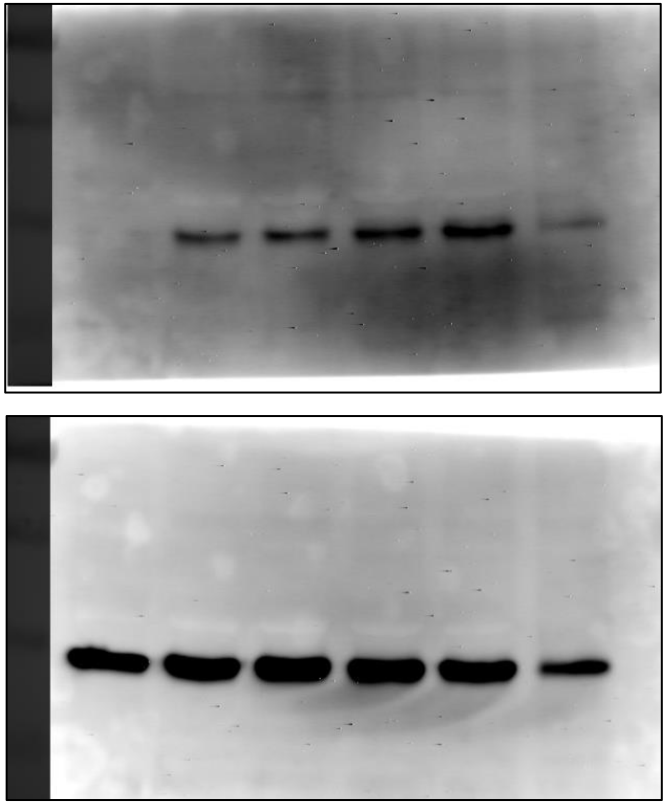

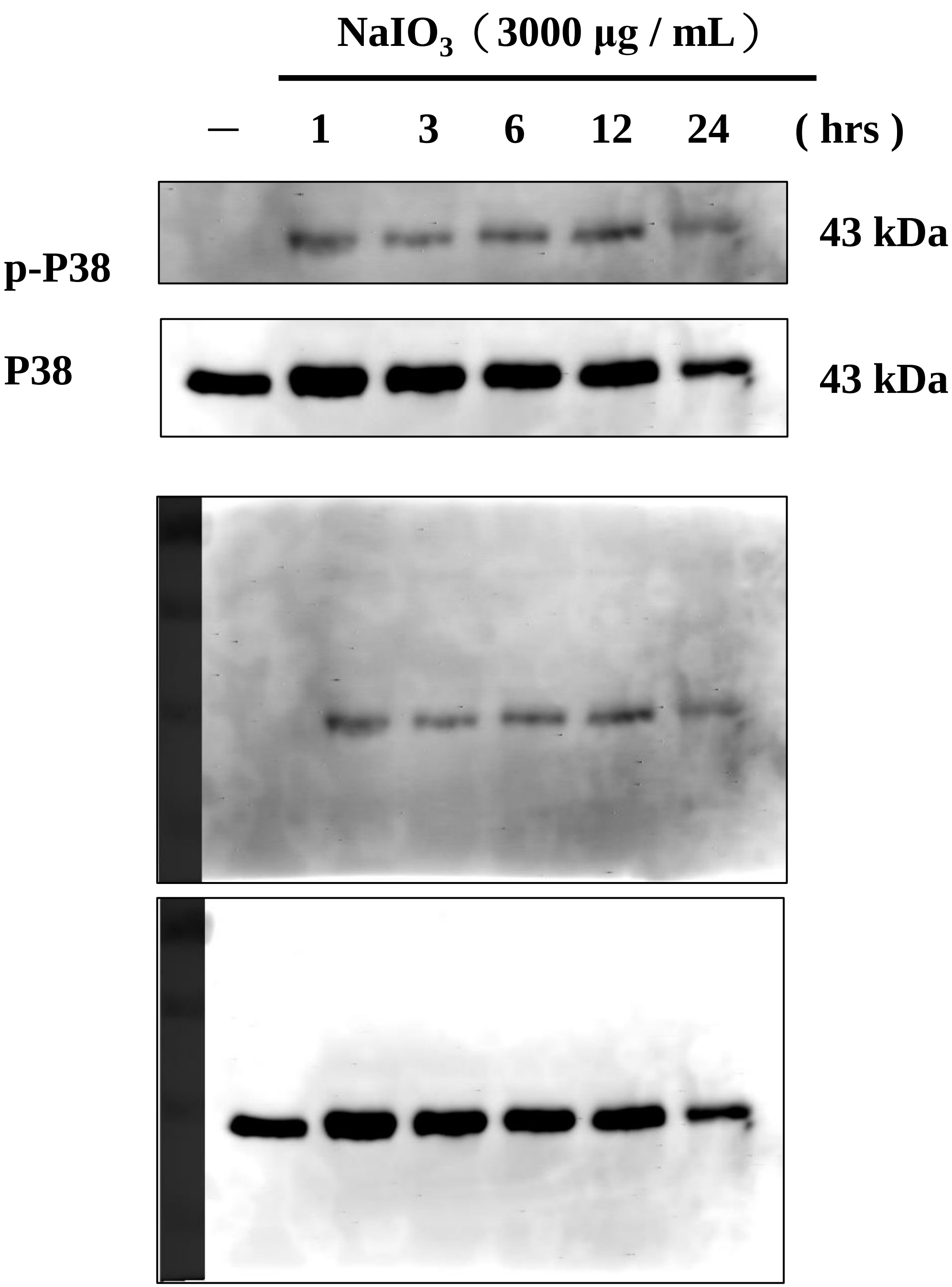
NaIO3（3000 µg / mL）
—
1
3
6
12
24
(hrs)
p-P38
43 kDa
P38
43 kDa

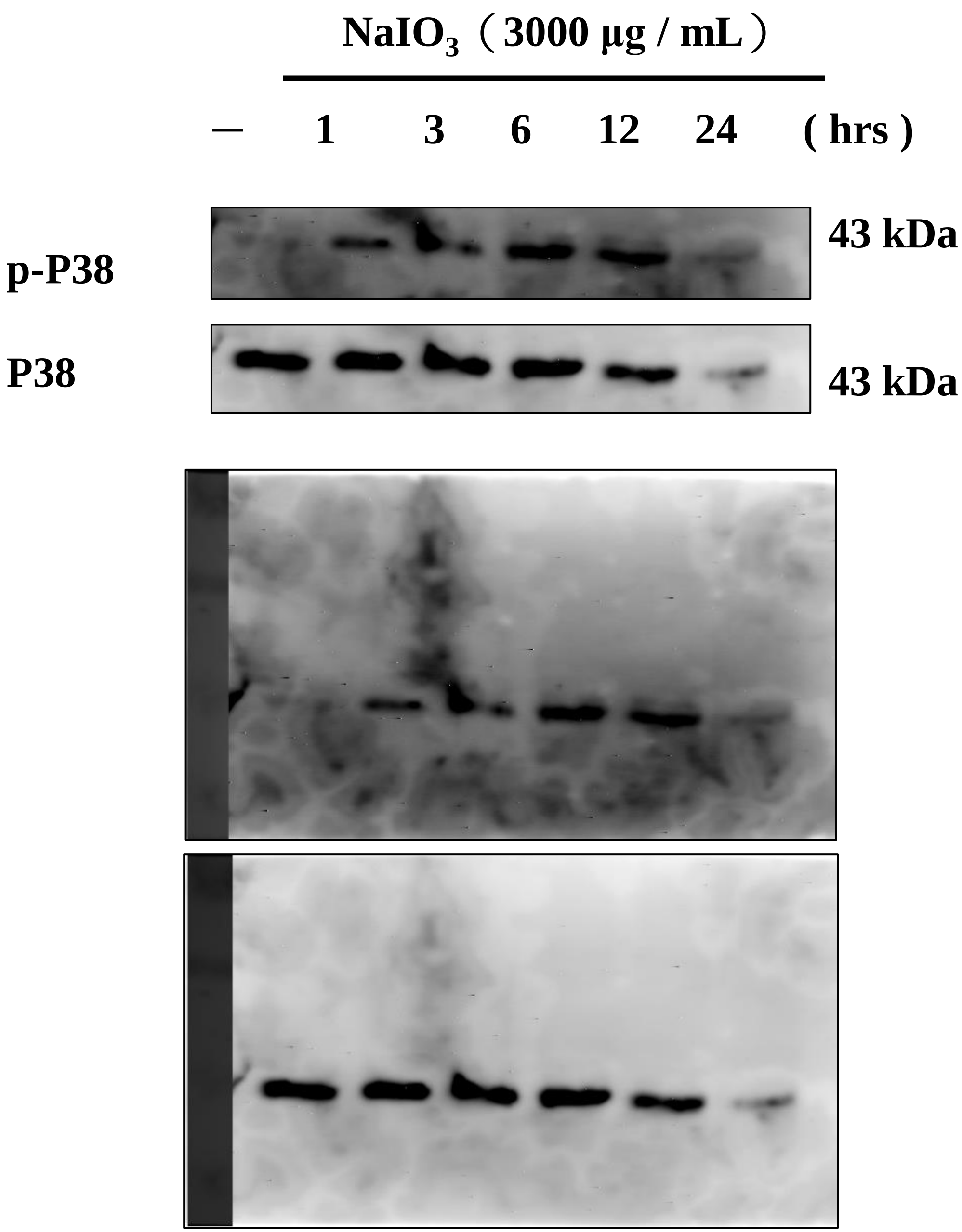

NaIO3（3000 µg / mL）
— 1 3 6 12 24 (hrs)
p-P38
43 kDa
P38
43 kDa

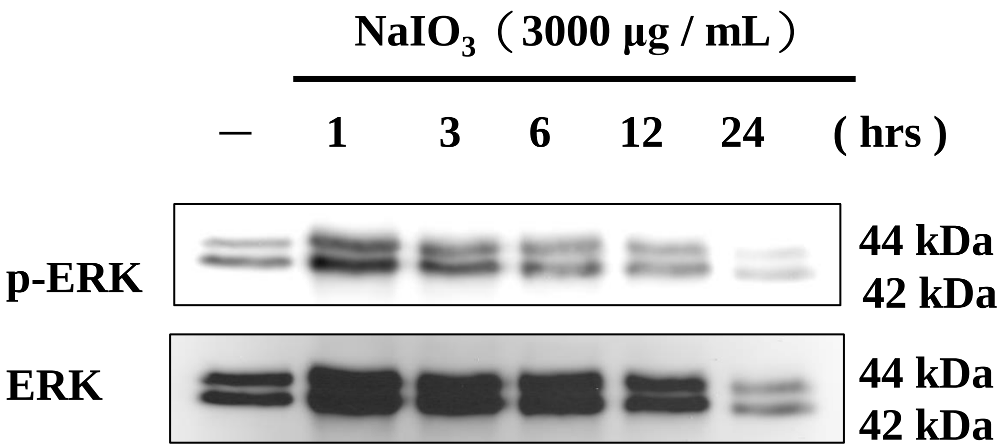
NaIO$_3$ (3000 μg / mL)
—
1
3
6
12
24
(hrs)
p-ERK
ERK
44 kDa
42 kDa
44 kDa
42 kDa

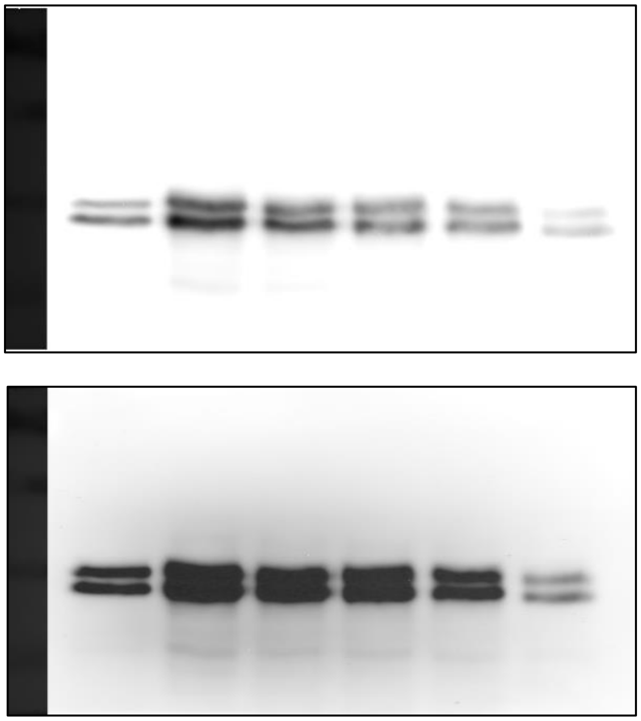

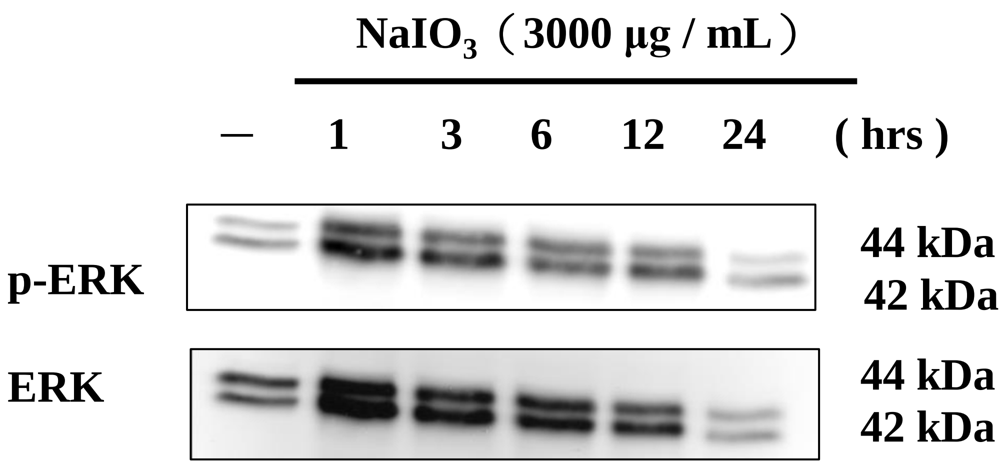
NaIO3（3000 μg / mL）
— 1 3 6 12 24 (hrs)
p-ERK
ERK
44 kDa
42 kDa
44 kDa
42 kDa

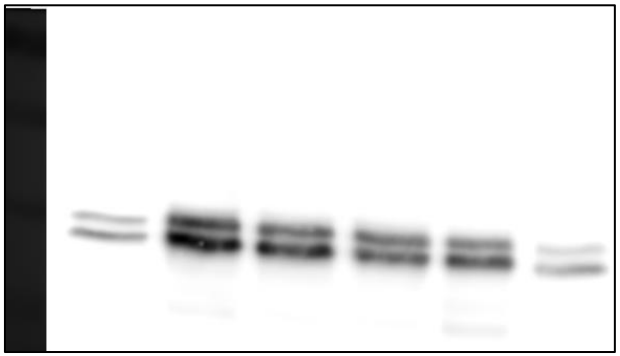

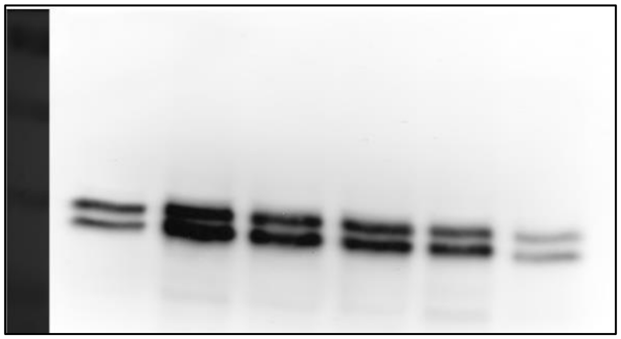

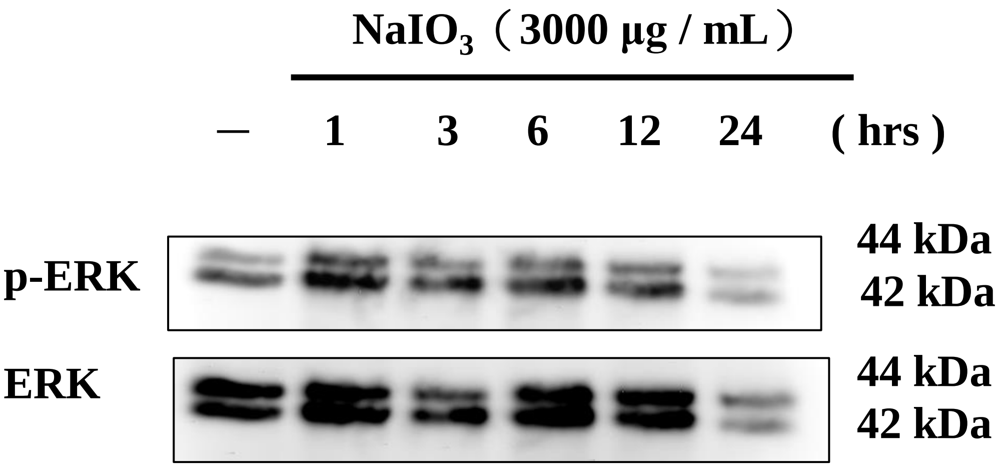
NaIO3（3000 μg / mL）
— 1 3 6 12 24 (hrs)
p-ERK
ERK
44 kDa
42 kDa
44 kDa
42 kDa

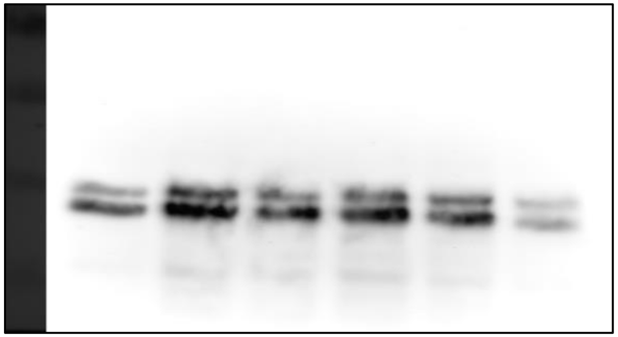

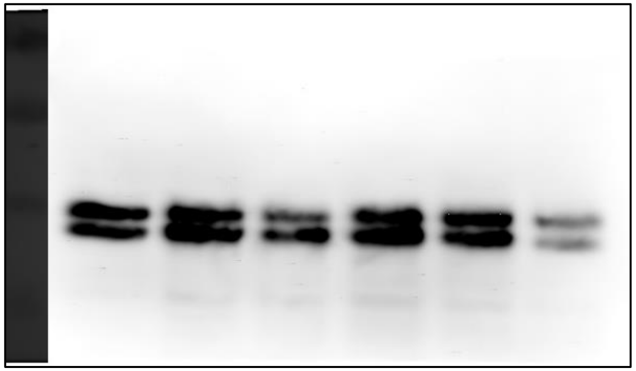

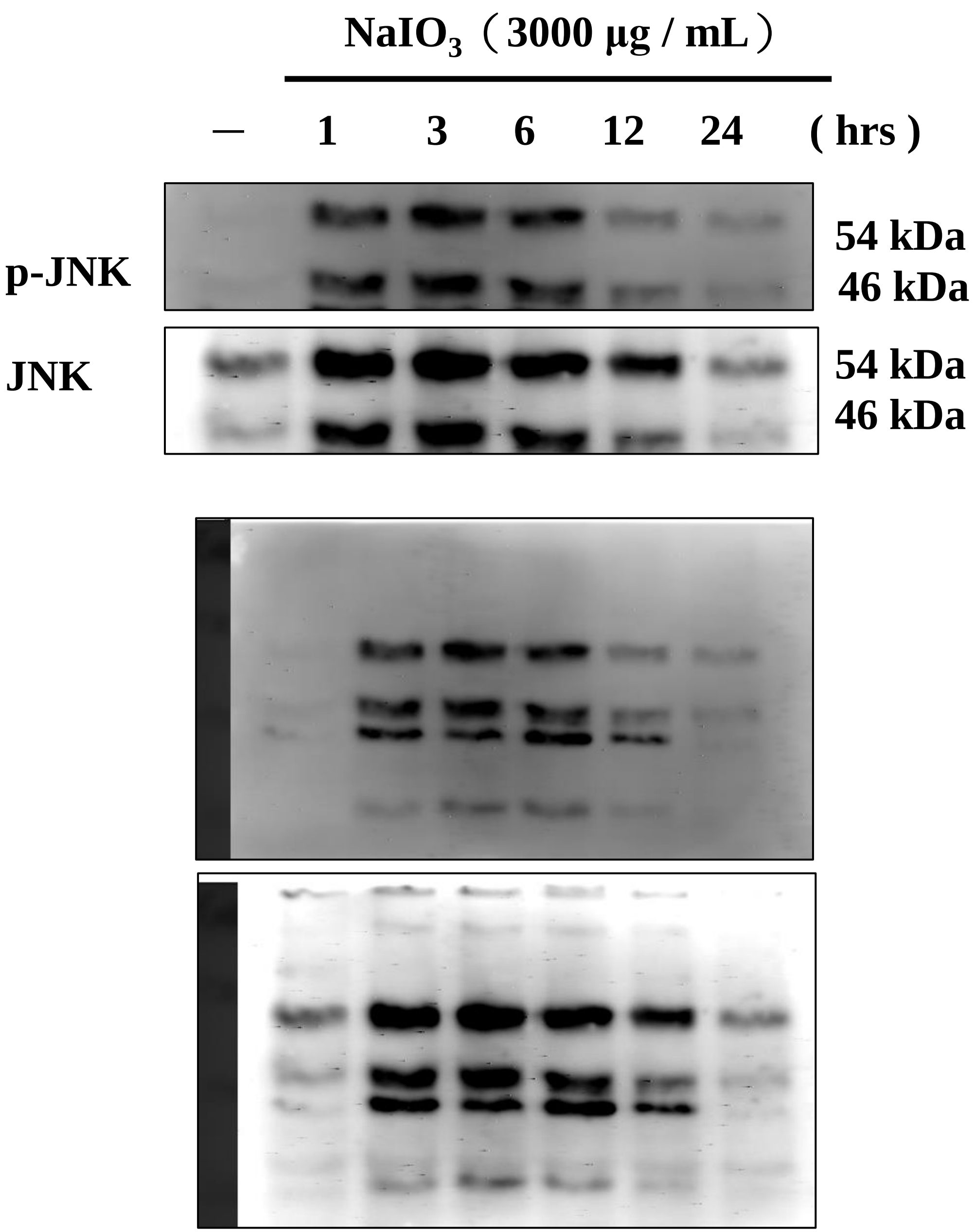
NaIO$_3$ (3000 µg / mL)
— 1 3 6 12 24 (hrs)
p-JNK
54 kDa
46 kDa
JNK
54 kDa
46 kDa

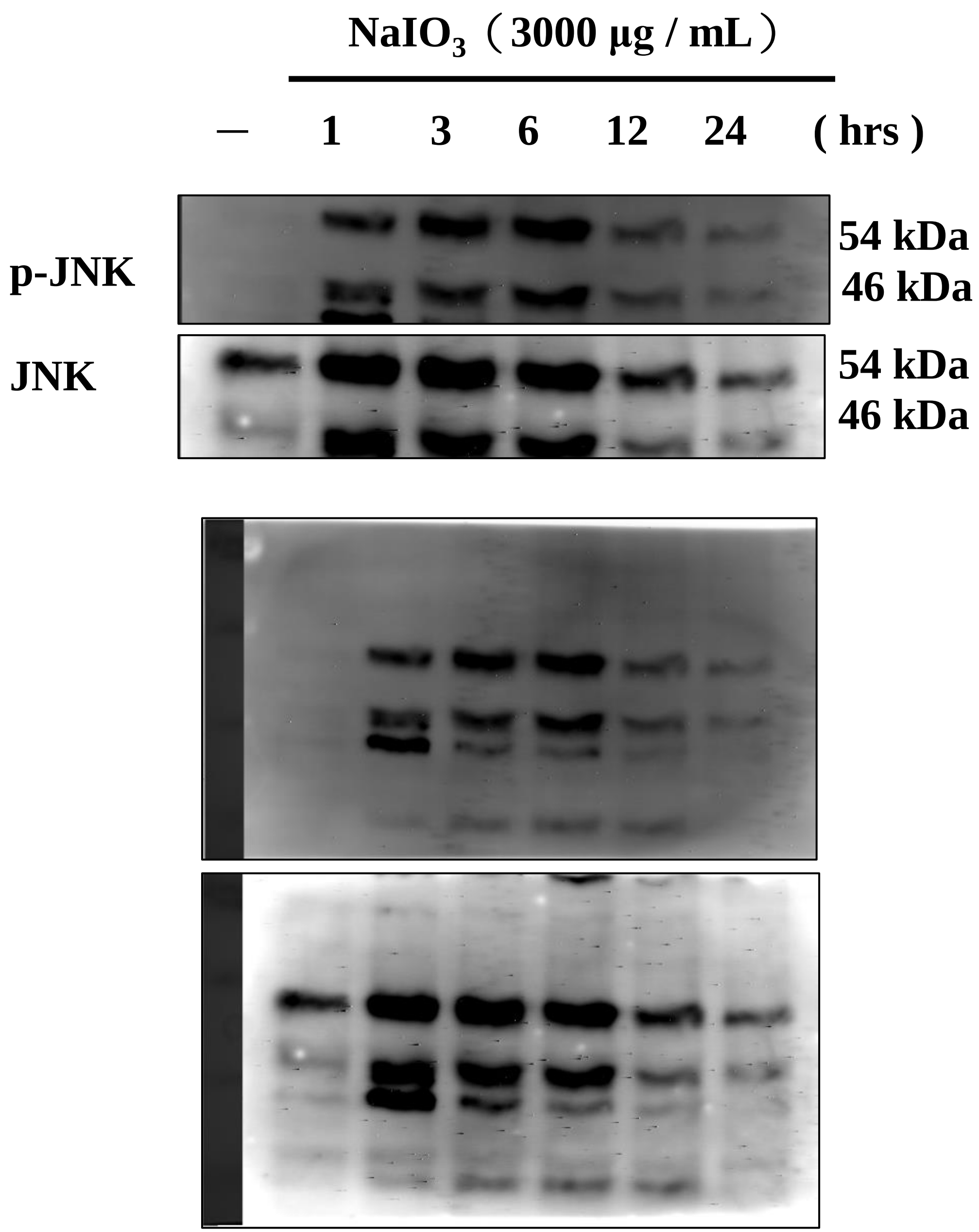

NaIO3（3000 µg / mL）
—
1
3
6
12
24
(hrs)
p-JNK
54 kDa
46 kDa
JNK
54 kDa
46 kDa

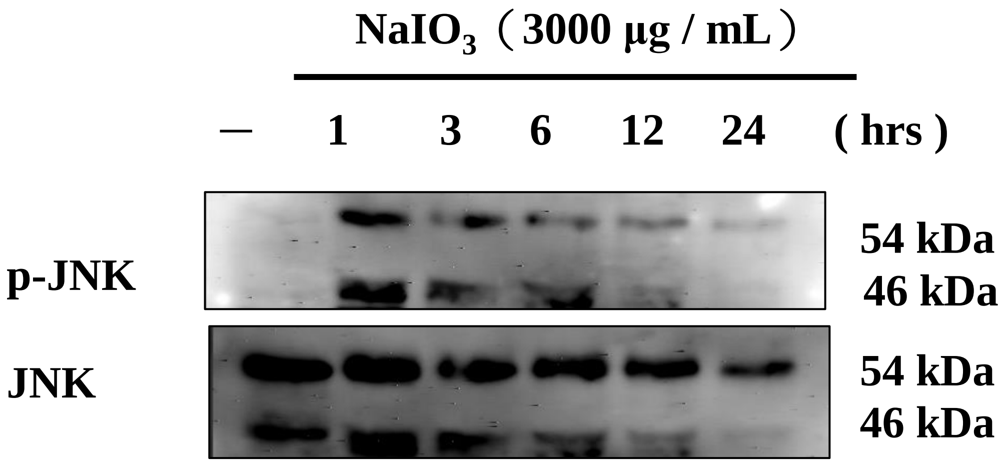
NaIO3（3000 μg / mL）
—
1
3
6
12
24
(hrs)
p-JNK
54 kDa
46 kDa
JNK
54 kDa
46 kDa

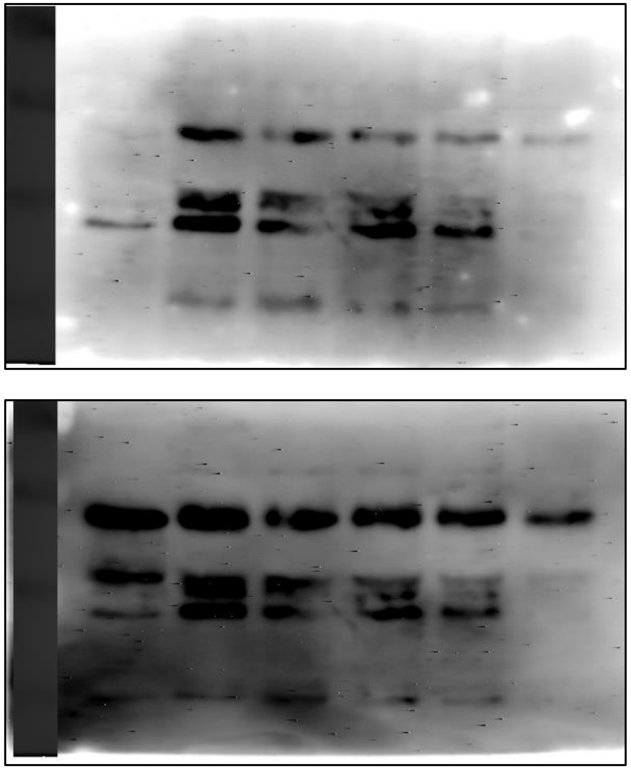

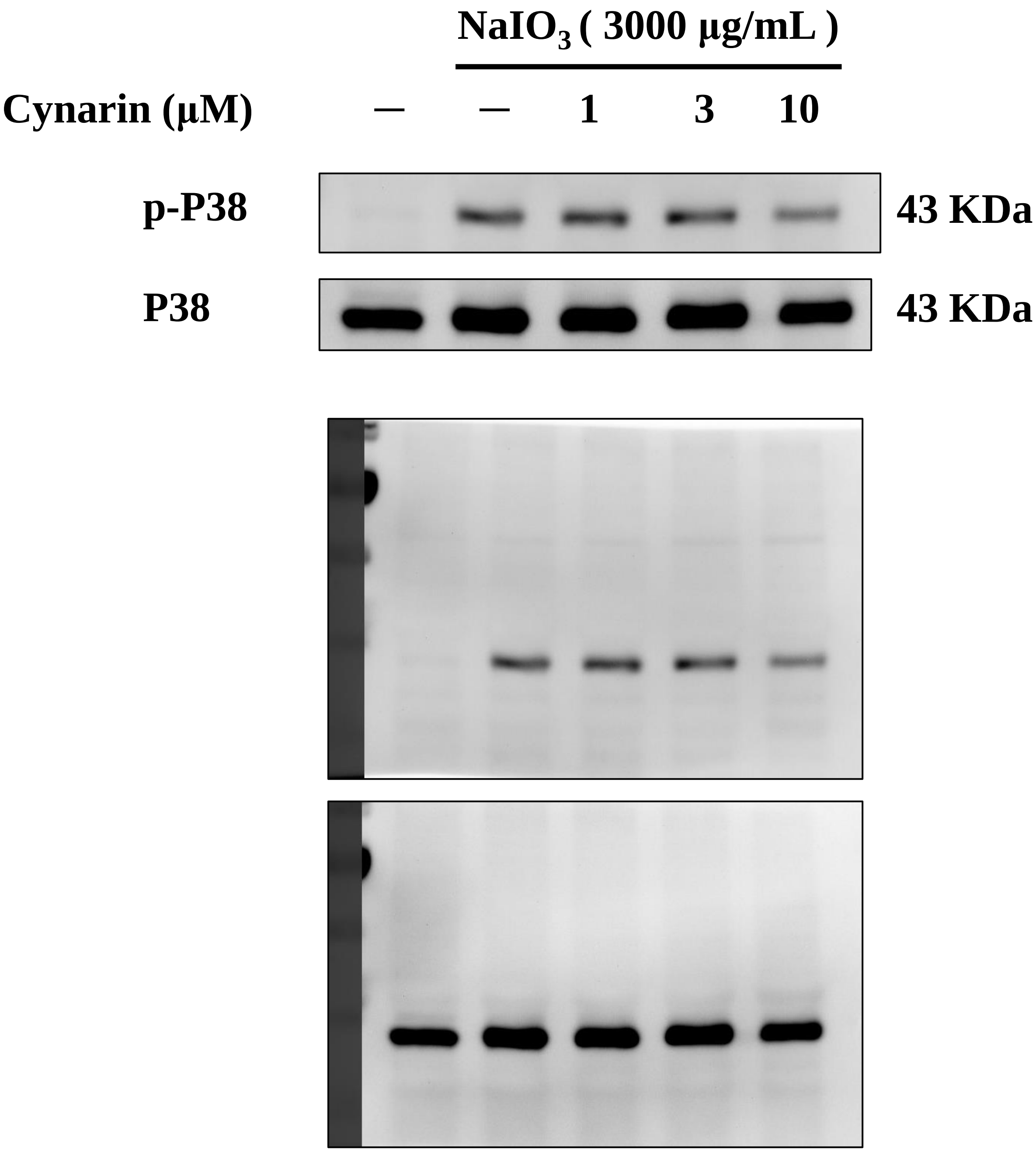
NaIO$_3$ (3000 µg/mL)
Cynarin (µM)
—
—
1
3
10
p-P38
43 KDa
P38
43 KDa

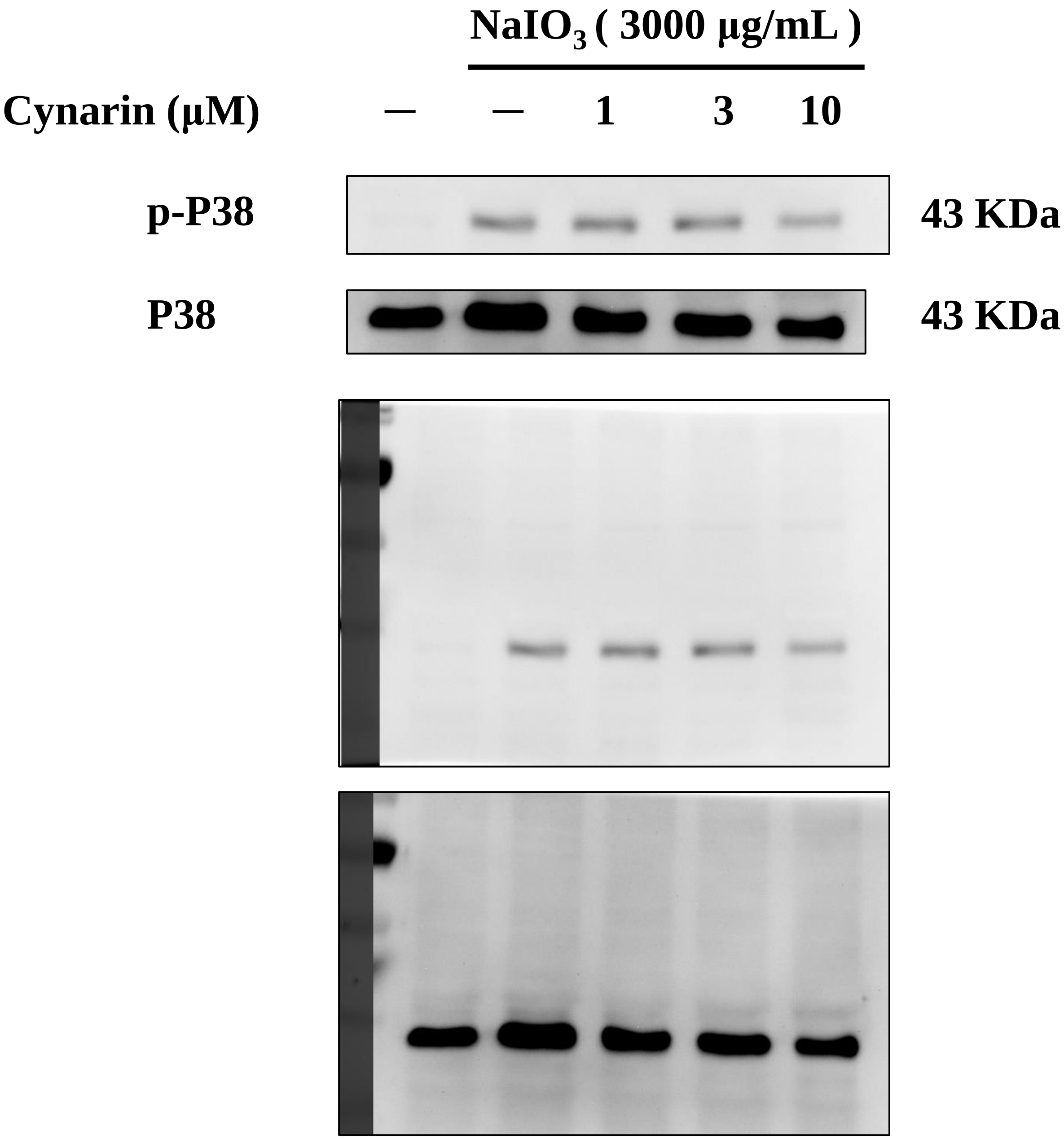
NaIO$_3$ (3000 µg/mL)
Cynarin (µM)
—
—
1
3
10
p-P38
43 KDa
P38
43 KDa

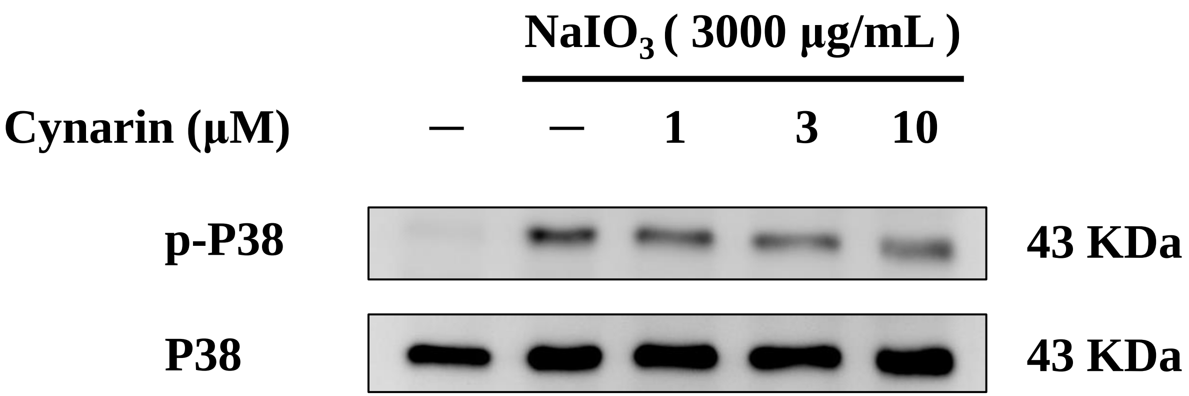

$NaIO_3$ (3000 µg/mL)
Cynarin (µM)
—
—
1
3
10
p-P38
P38
43 KDa
43 KDa

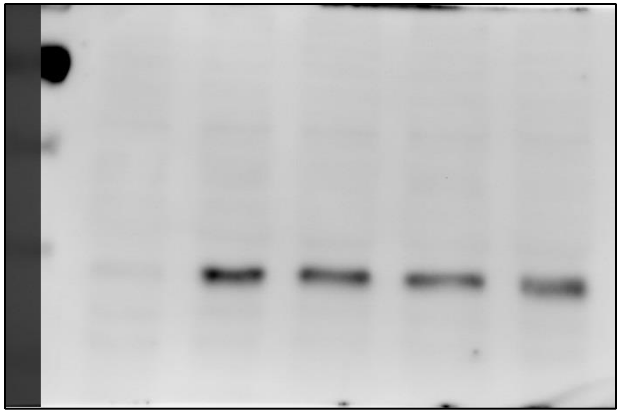

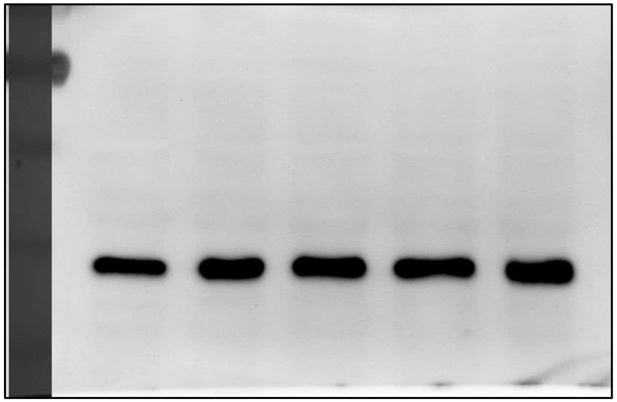

Cynarin (μM) — 1 3 10

p-P38 43 KDa

P38 43 KDa

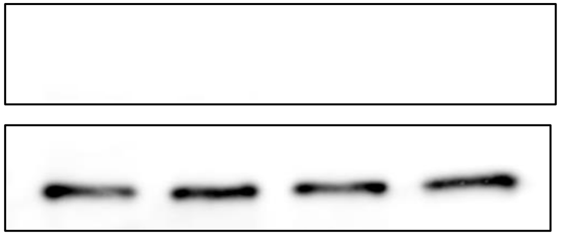

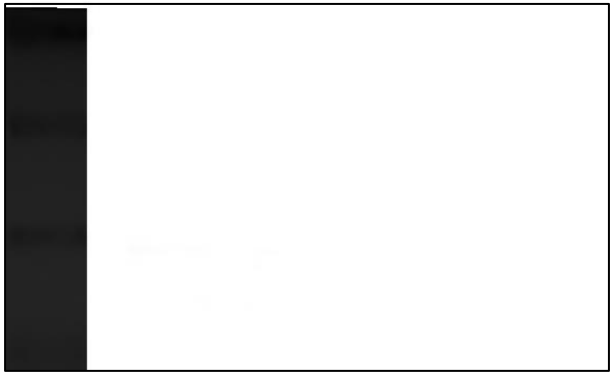

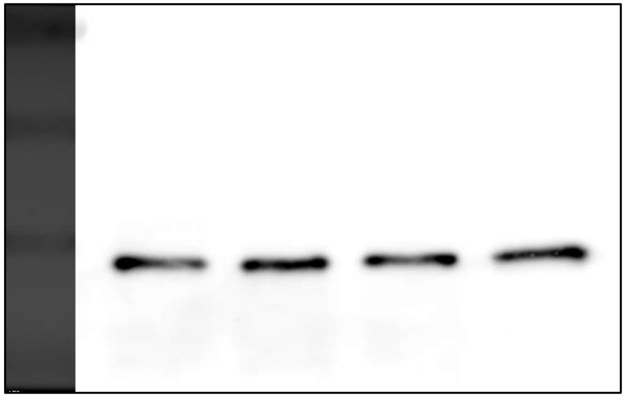

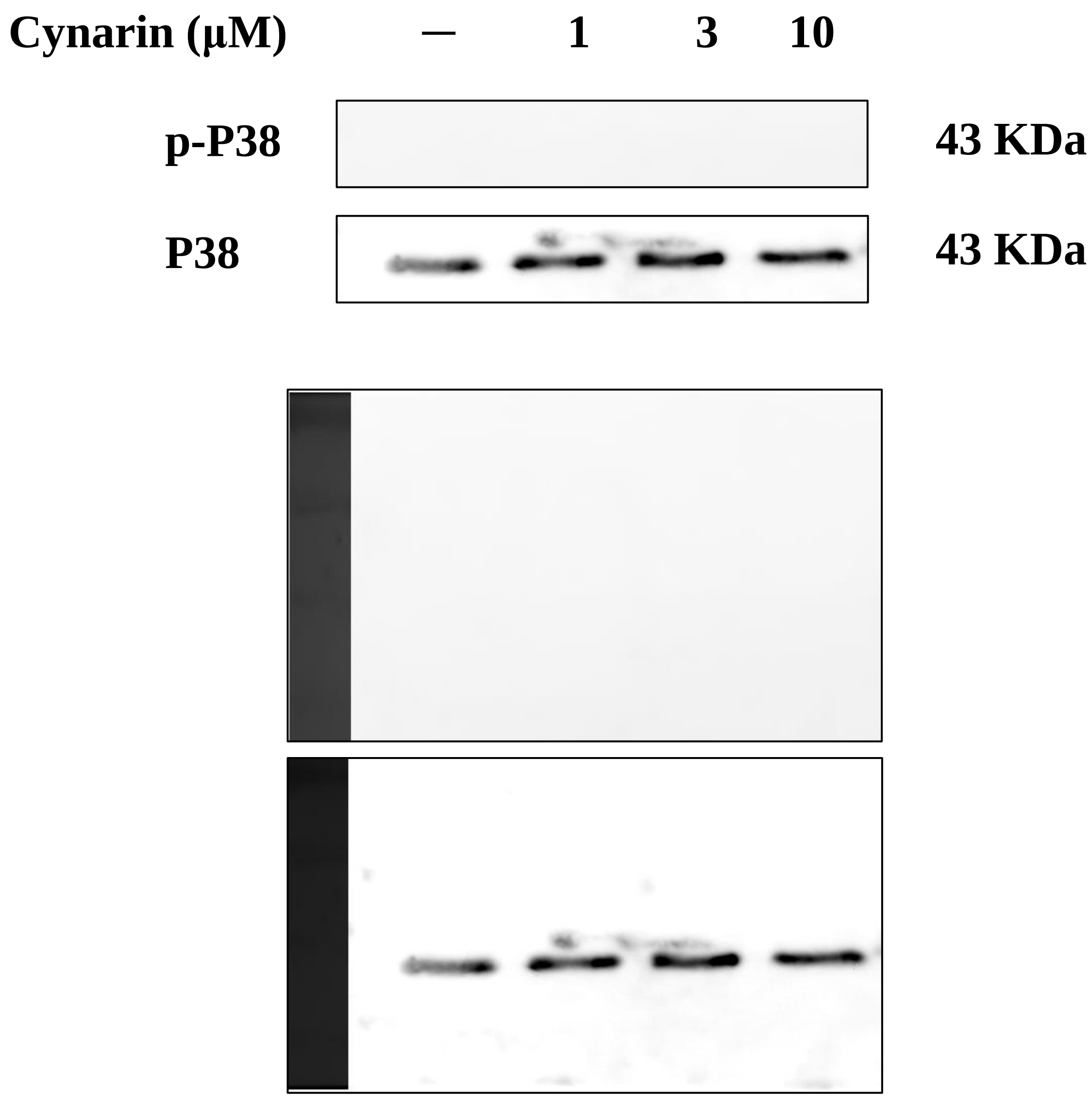
Cynarin (μM)
—
1
3
10
p-P38
43 KDa
P38
43 KDa

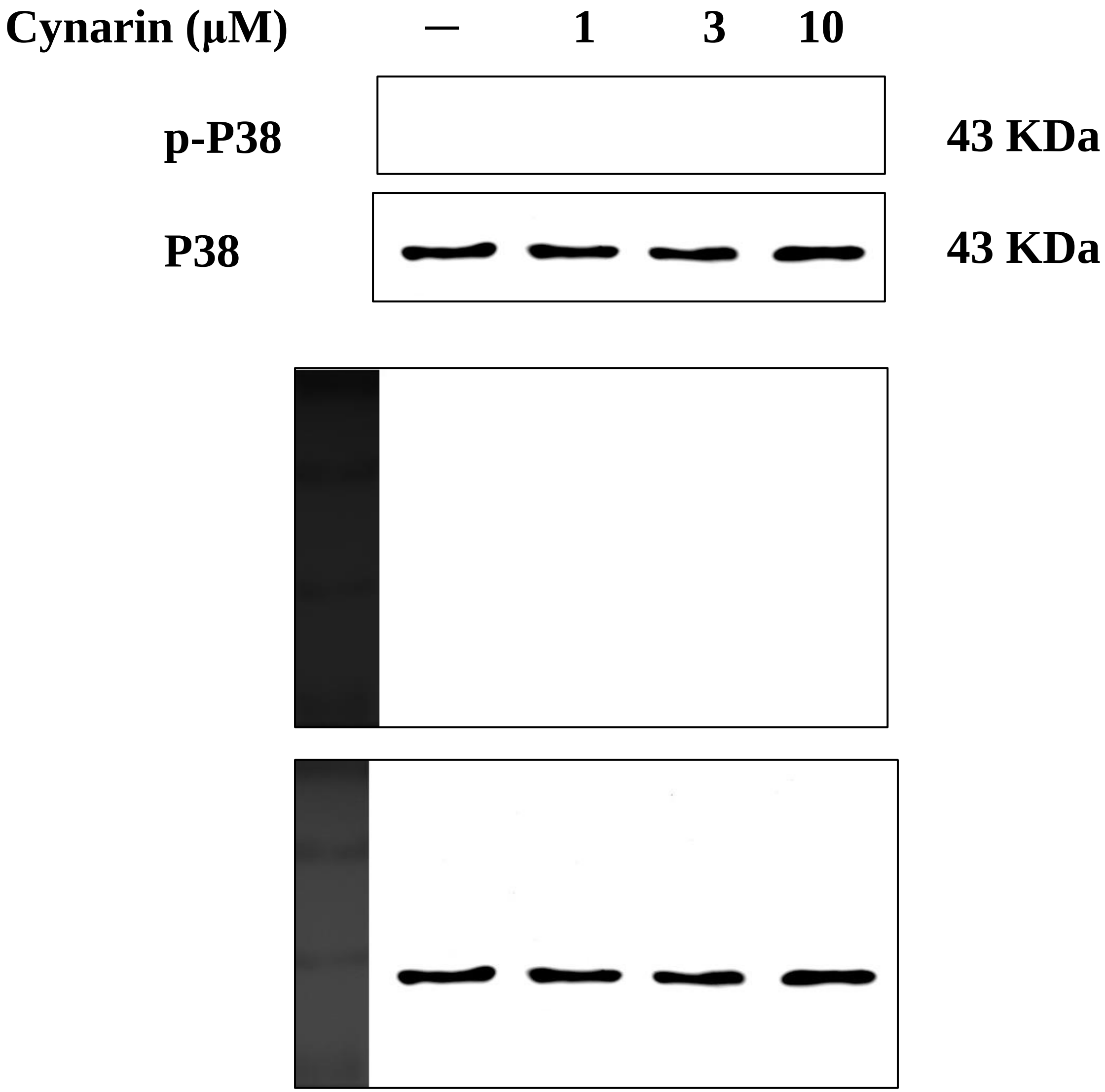

Cynarin (μM)
—
1
3
10
p-P38
43 KDa
P38
43 KDa

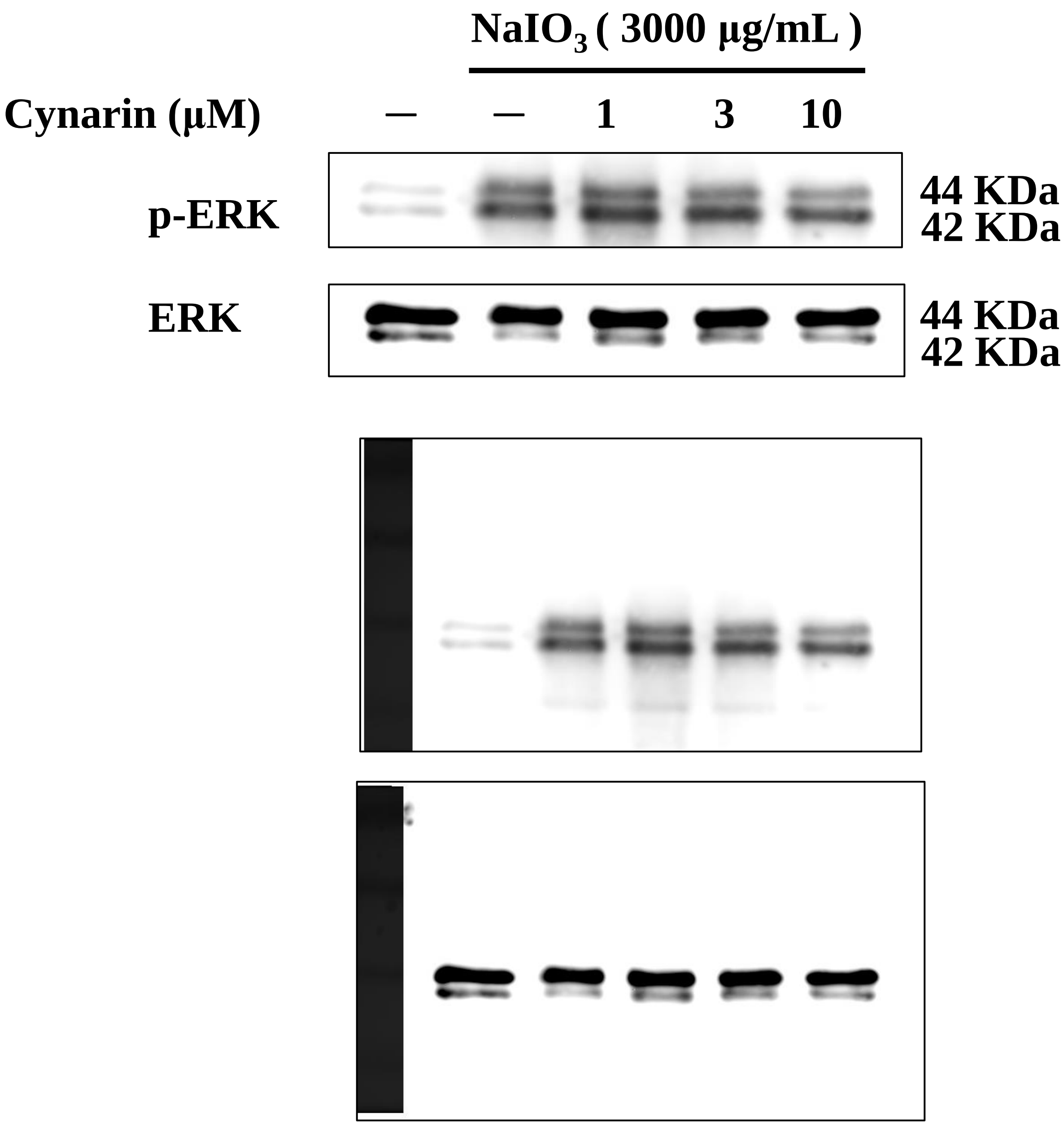
NaIO3 (3000 µg/mL)
Cynarin (µM)
—
—
1
3
10
p-ERK
44 KDa
42 KDa
ERK
44 KDa
42 KDa

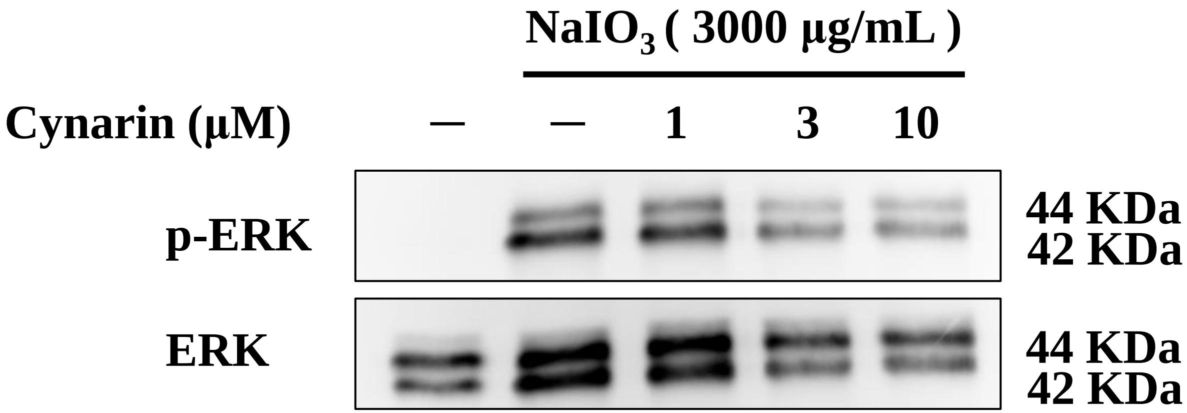
NaIO3 (3000 µg/mL)
Cynarin (µM)
—
—
1
3
10
p-ERK
ERK
44 KDa
42 KDa
44 KDa
42 KDa

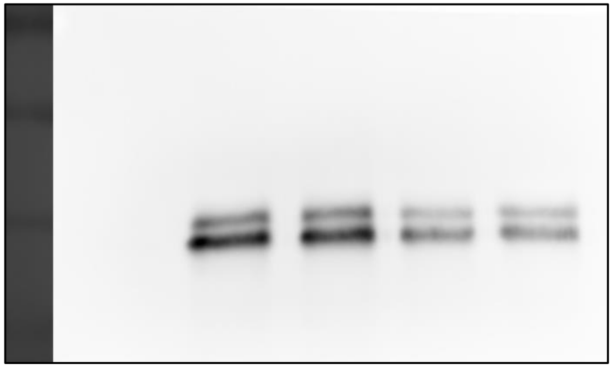

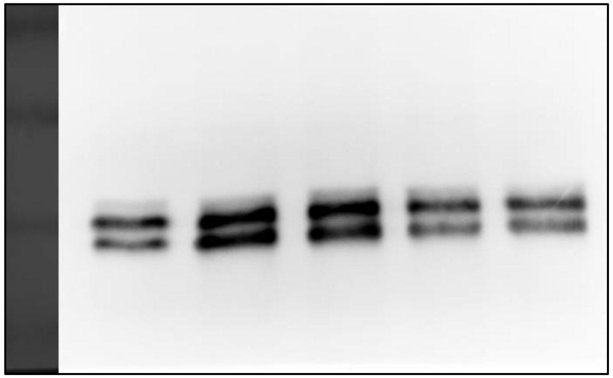

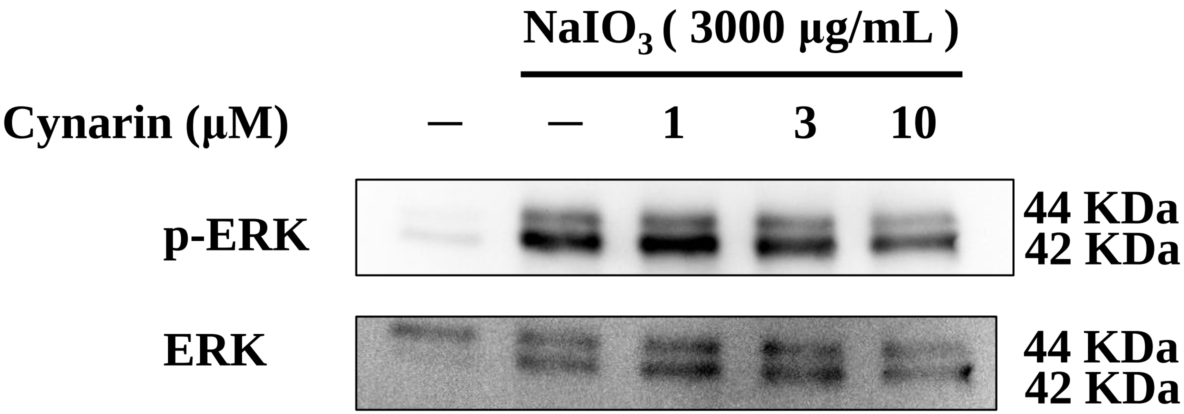
NaIO3 (3000 µg/mL)
Cynarin (µM)
—
—
1
3
10
p-ERK
44 KDa
42 KDa
ERK
44 KDa
42 KDa

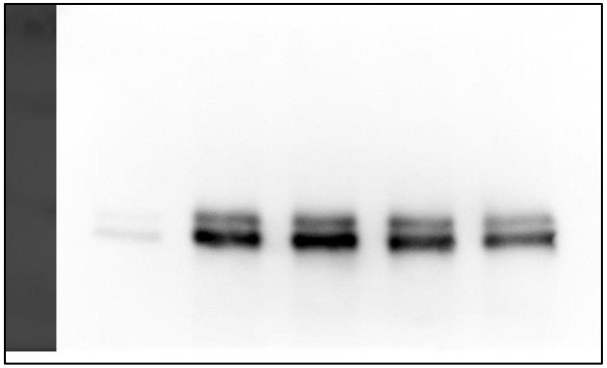

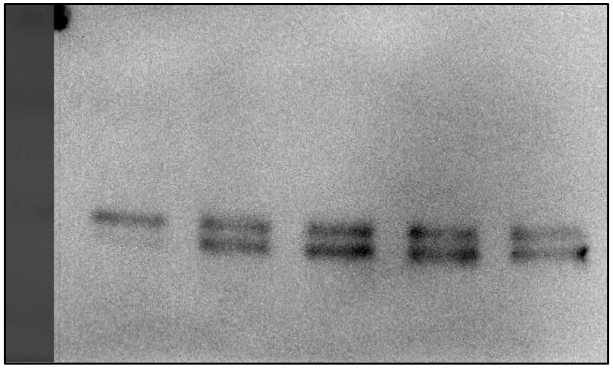

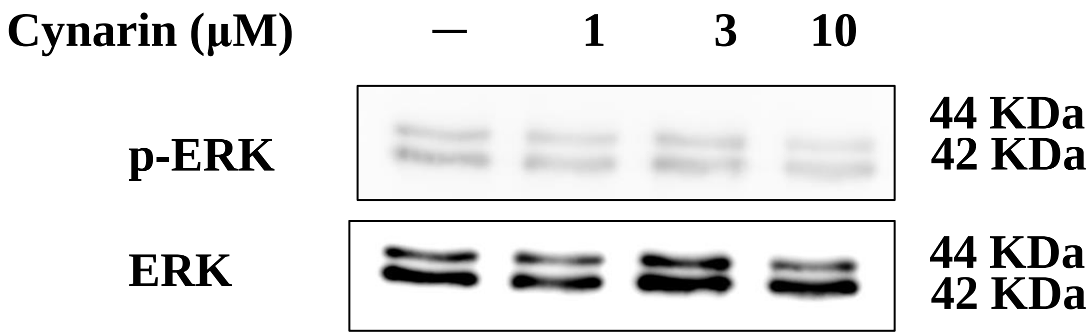
Cynarin (μM)
—
1
3
10
p-ERK
44 KDa
42 KDa
ERK
44 KDa
42 KDa

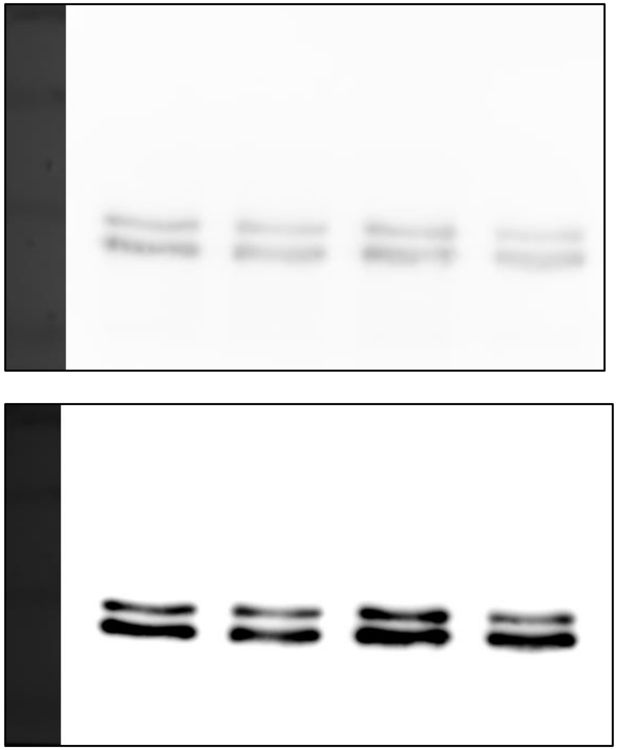

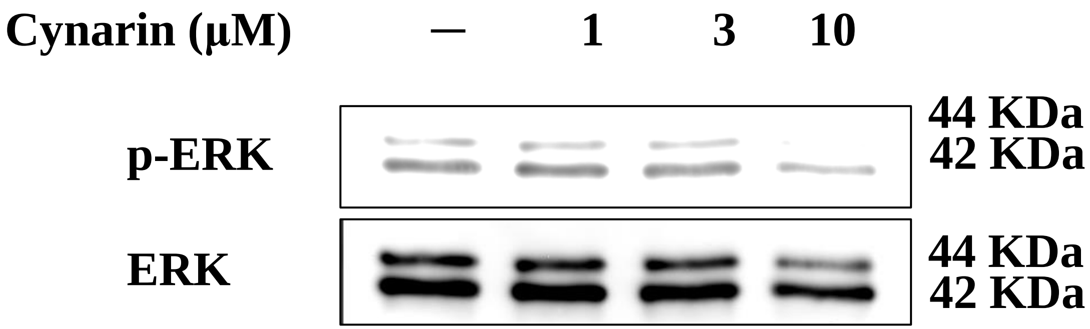
Cynarin (μM)
—
1
3
10
p-ERK
44 KDa
42 KDa
ERK
44 KDa
42 KDa

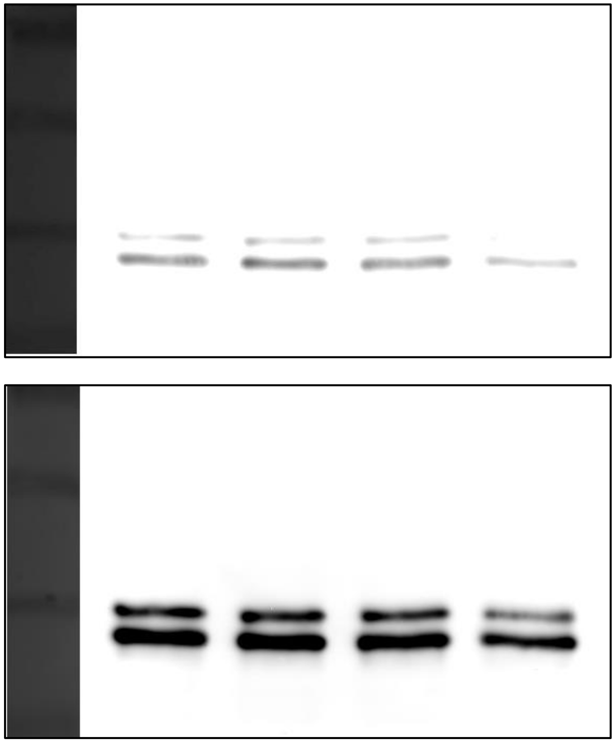

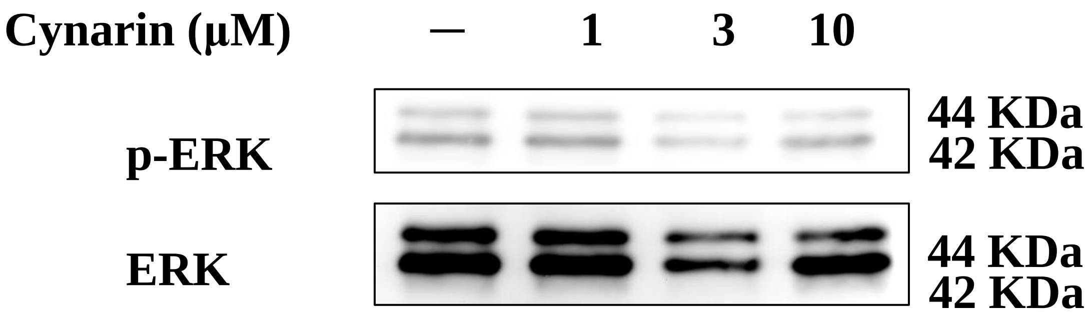
Cynarin (μM)
—
1
3
10
p-ERK
44 KDa
42 KDa
ERK
44 KDa
42 KDa

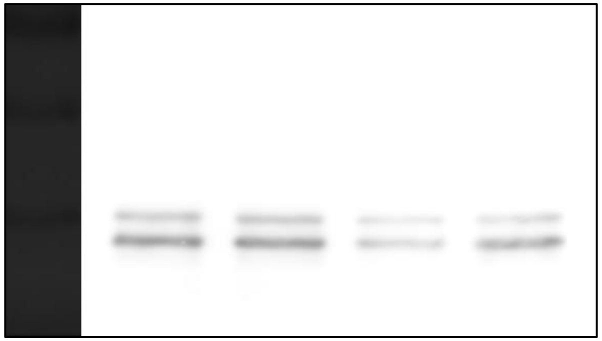

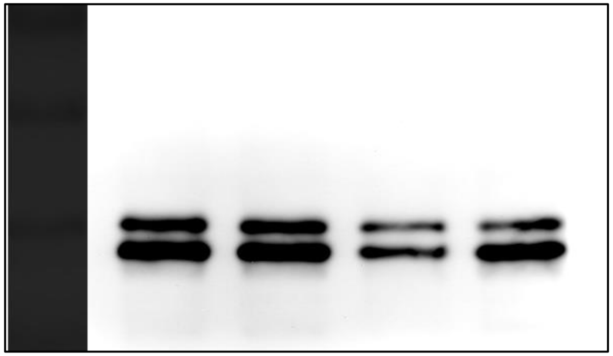

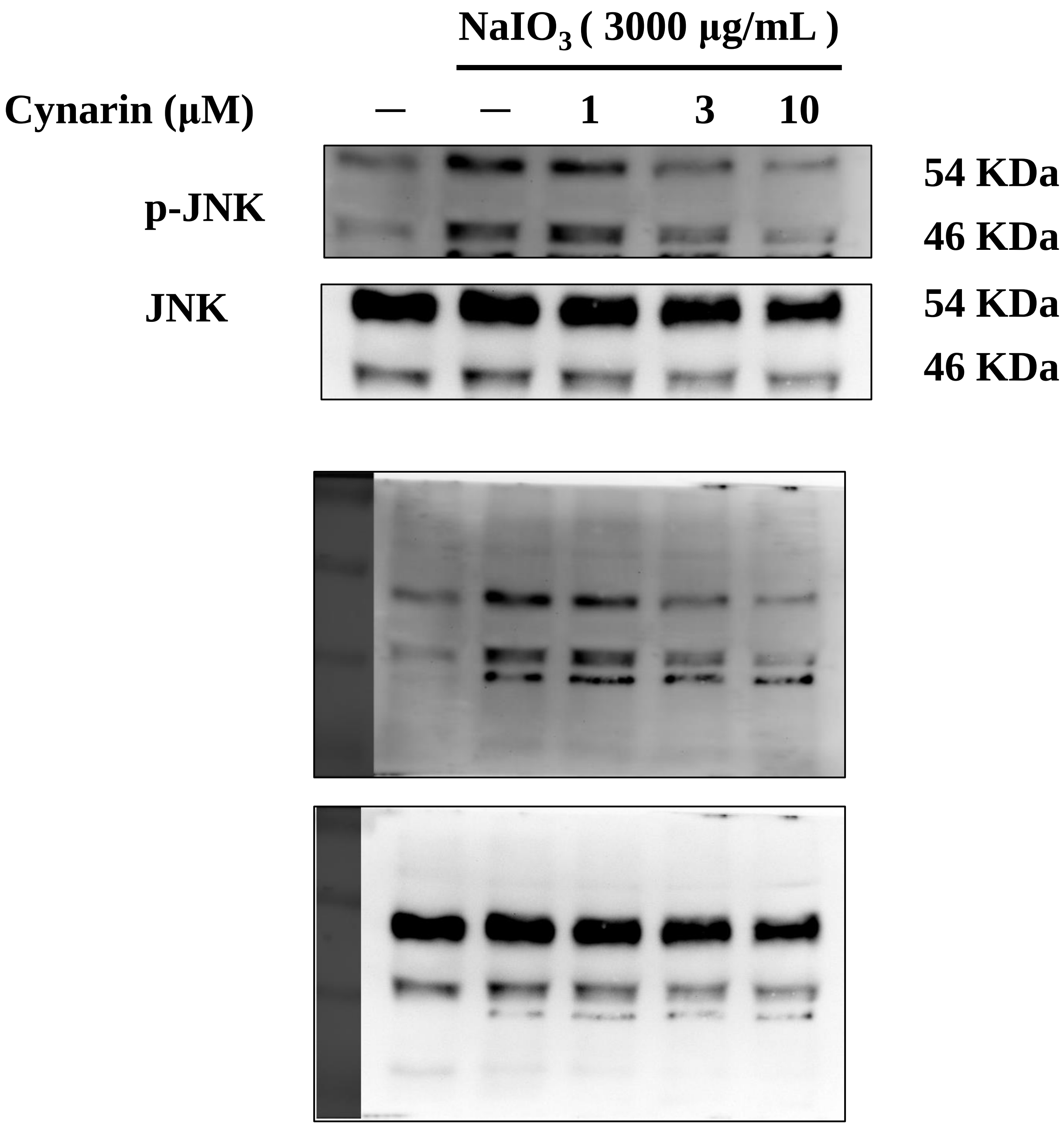
NaIO3 (3000 µg/mL)
Cynarin (µM)
—
—
1
3
10
p-JNK
JNK
54 KDa
46 KDa
54 KDa
46 KDa

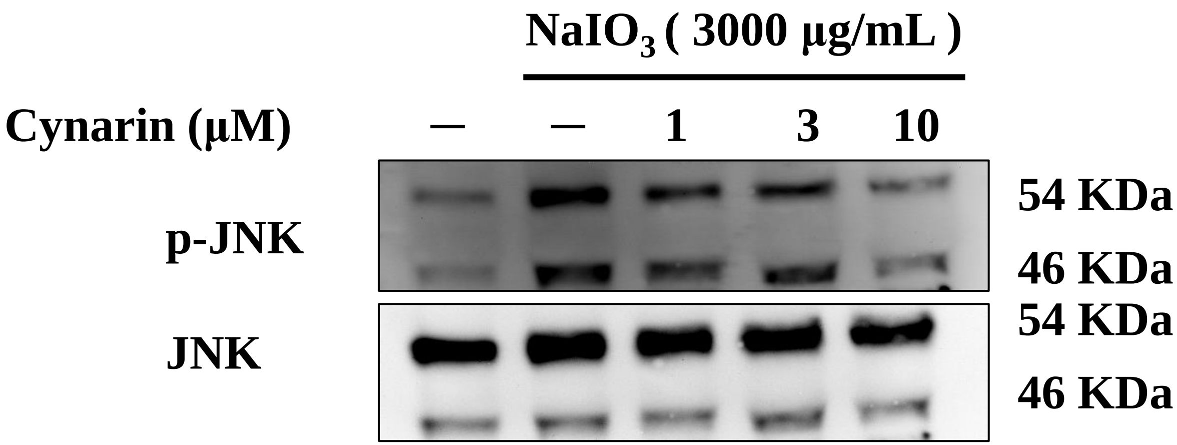
NaIO3 (3000 µg/mL)
Cynarin (µM)
—
—
1
3
10
p-JNK
JNK
54 KDa
46 KDa
54 KDa
46 KDa

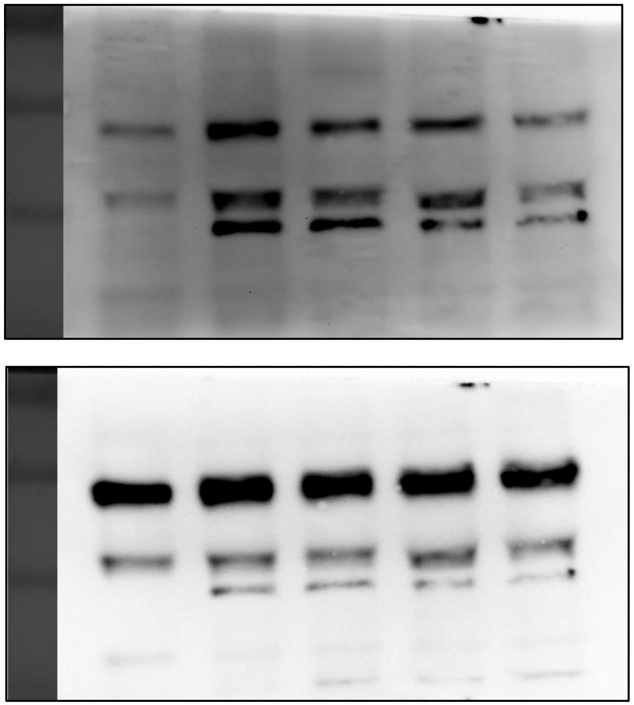

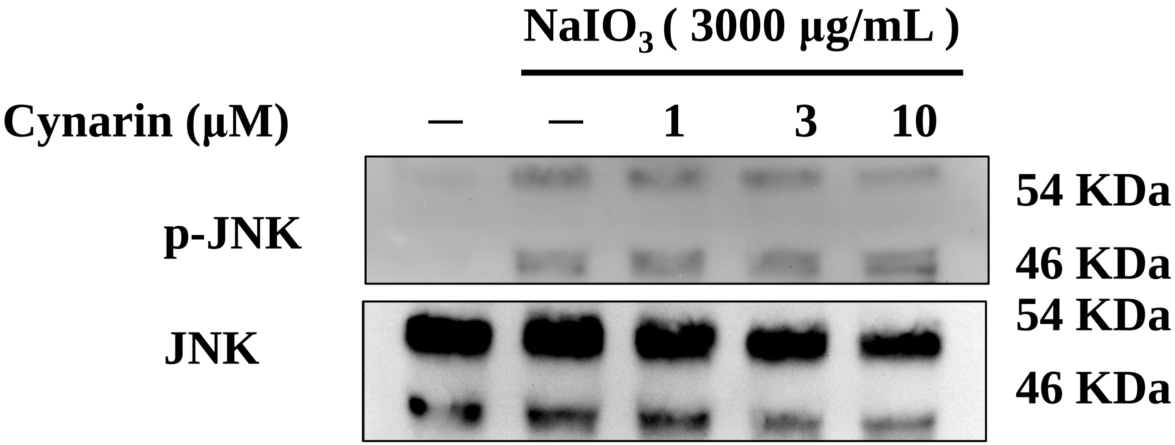
NaIO$_3$ (3000 µg/mL)
Cynarin (µM)
—
—
1
3
10
p-JNK
JNK
54 KDa
46 KDa
54 KDa
46 KDa

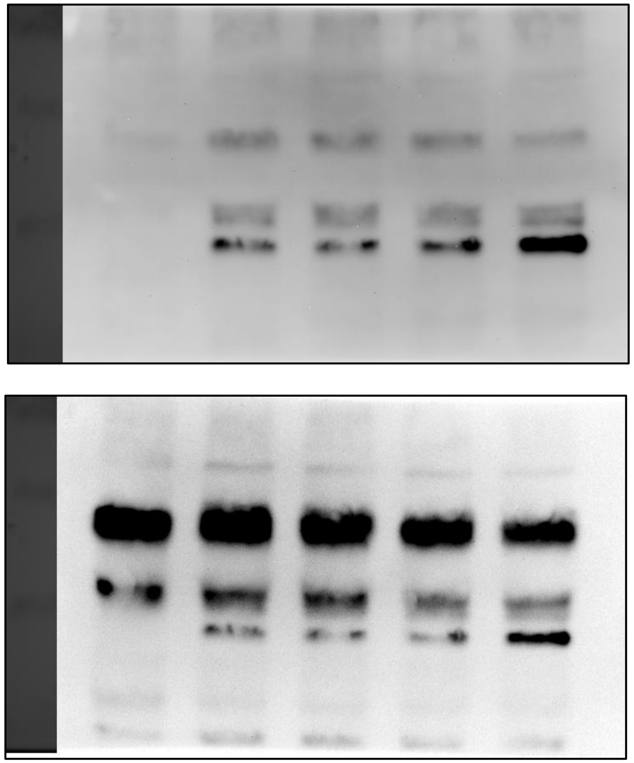

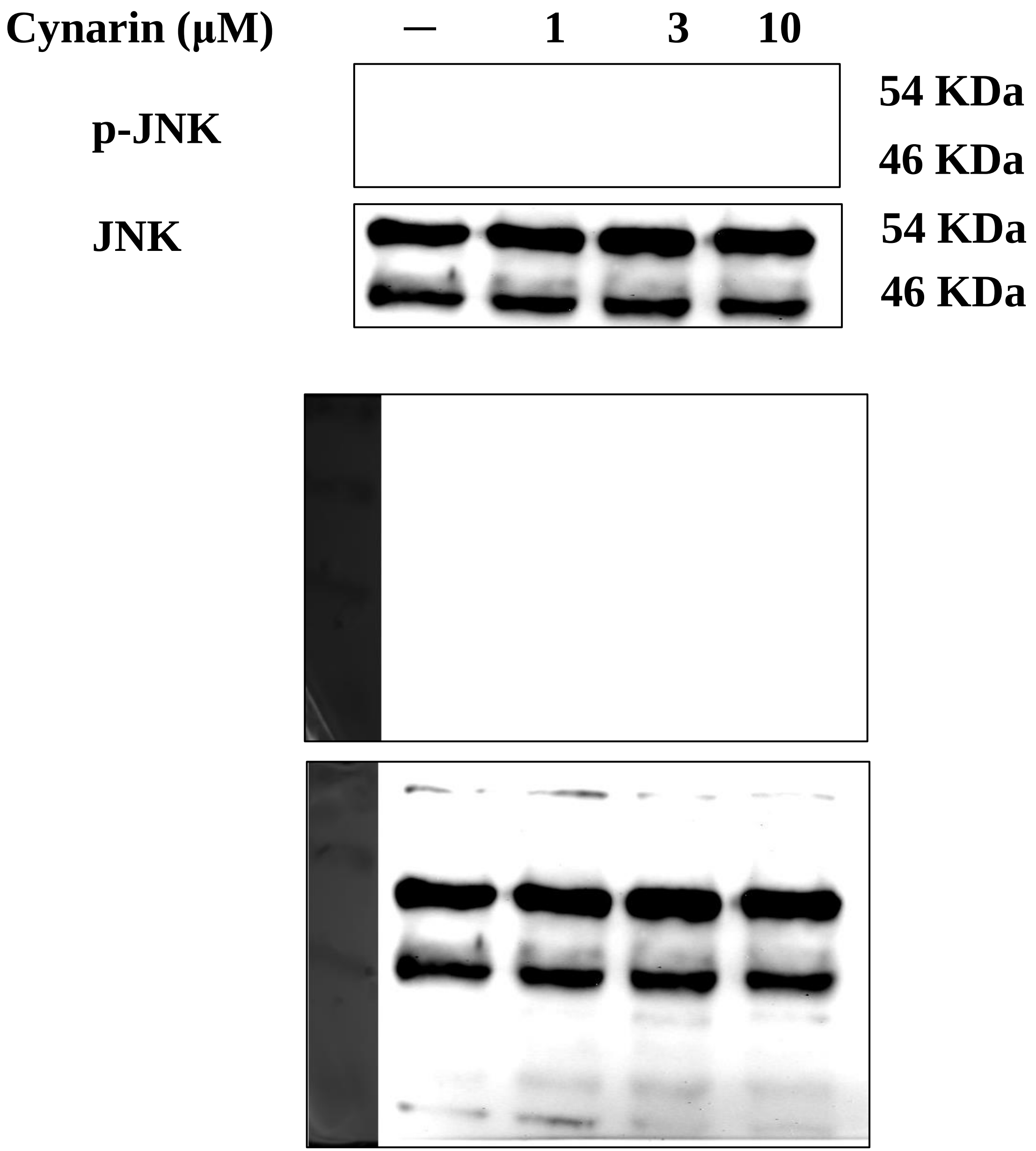
Cynarin (μM)
—
1
3
10
p-JNK
54 KDa
46 KDa
JNK
54 KDa
46 KDa

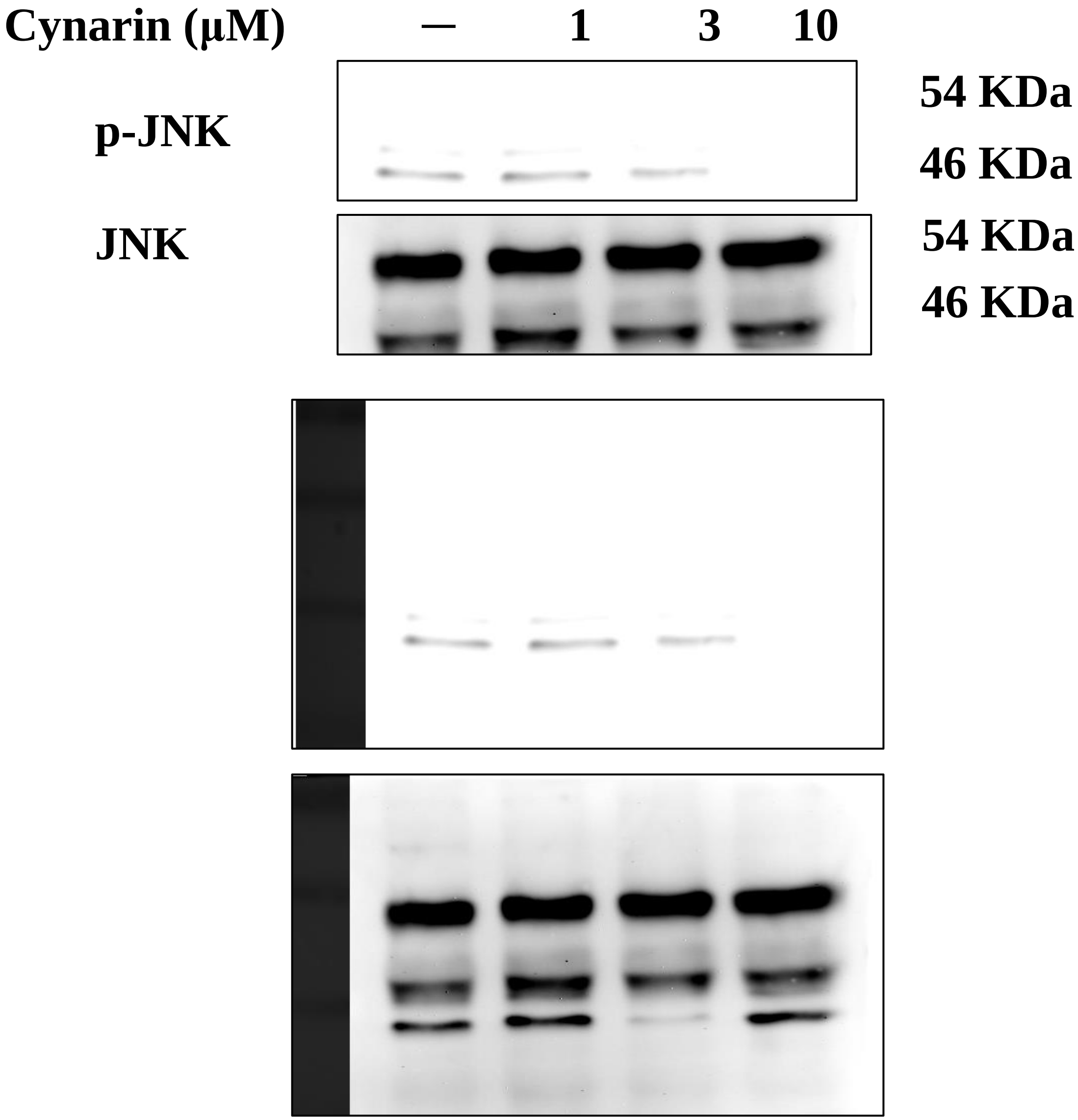

Cynarin (μM) — 1 3 10
p-JNK
54 KDa
46 KDa
JNK
54 KDa
46 KDa

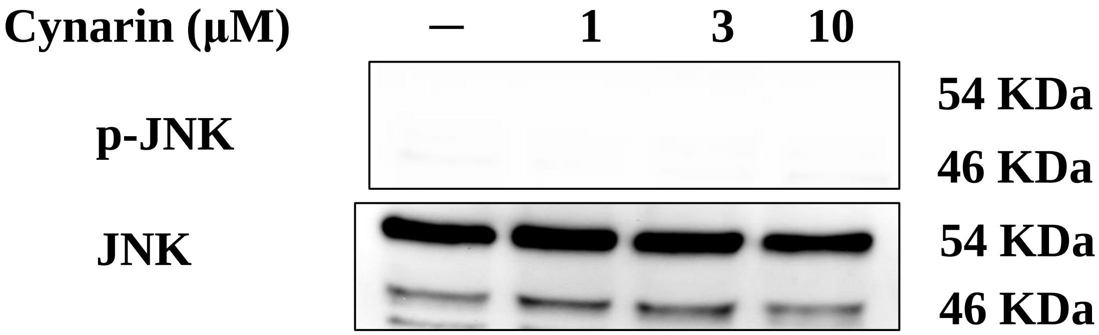
Cynarin (μM)
—
1
3
10
p-JNK
54 KDa
46 KDa
JNK
54 KDa
46 KDa

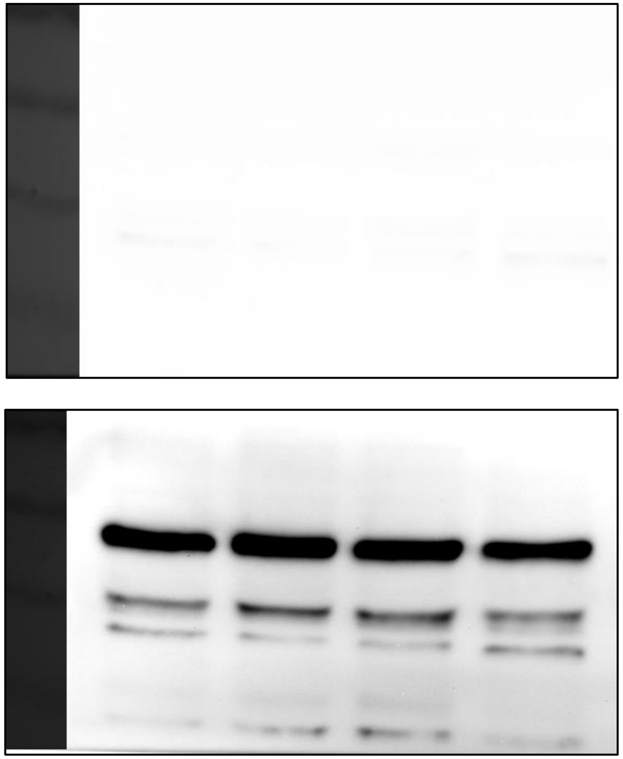

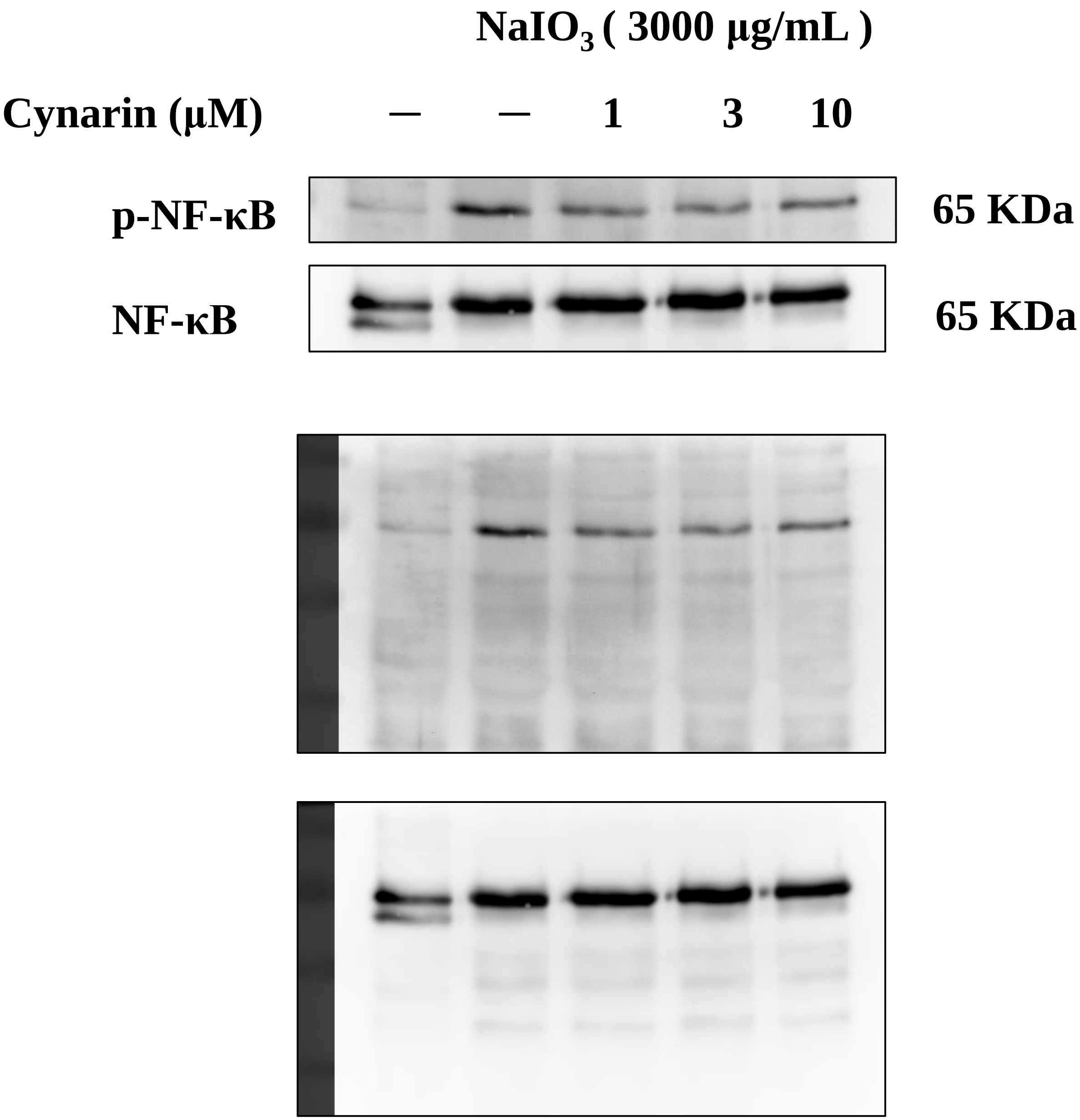
NaIO3 (3000 µg/mL)
Cynarin (µM)
—
—
1
3
10
p-NF-κB
65 KDa
NF-κB
65 KDa

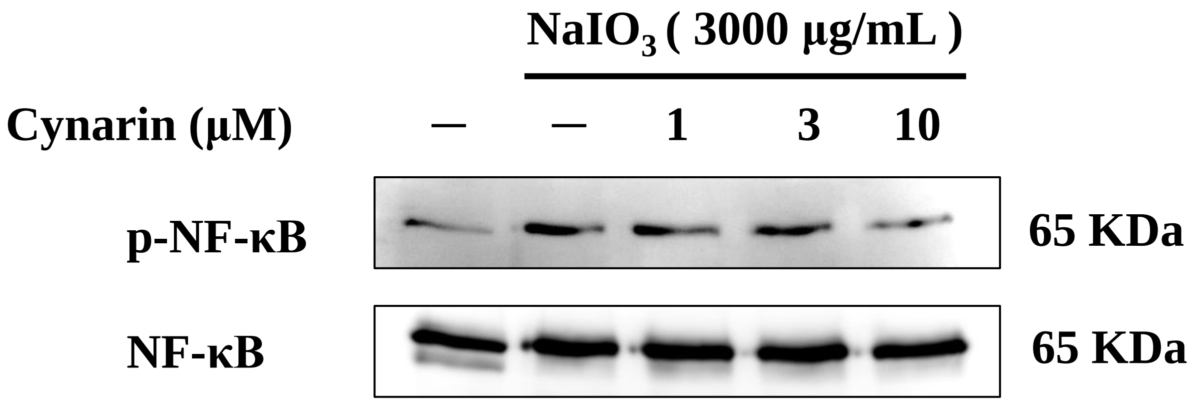
NaIO3 (3000 µg/mL)
Cynarin (µM)
—
—
1
3
10
p-NF-κB
NF-κB
65 KDa
65 KDa

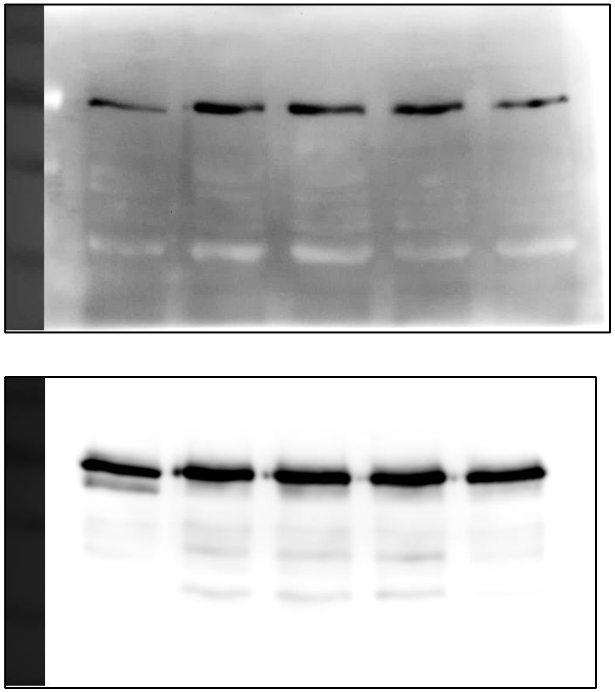

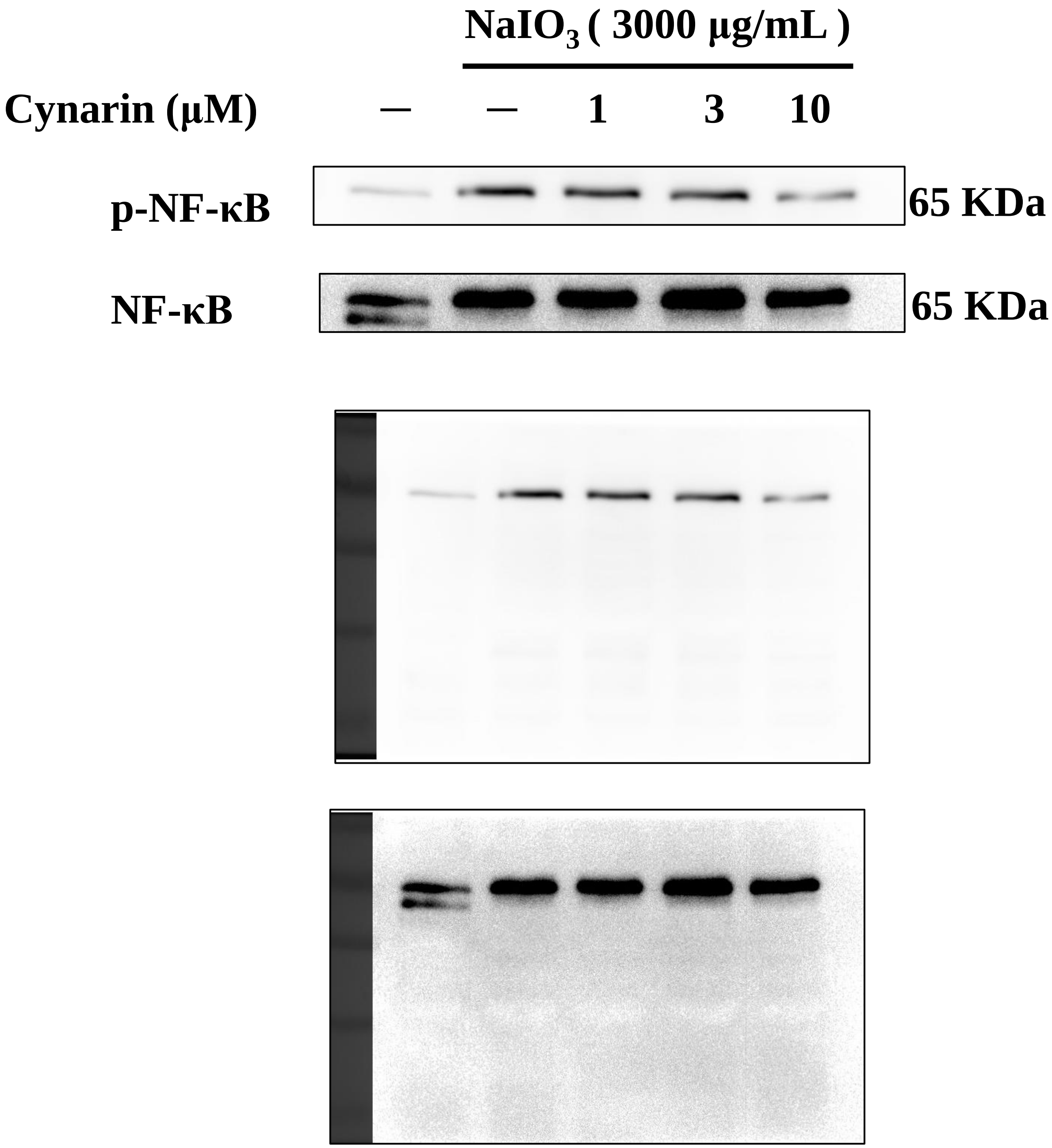
NaIO3 (3000 µg/mL)
Cynarin (µM)
—
—
1
3
10
p-NF-κB
65 KDa
NF-κB
65 KDa

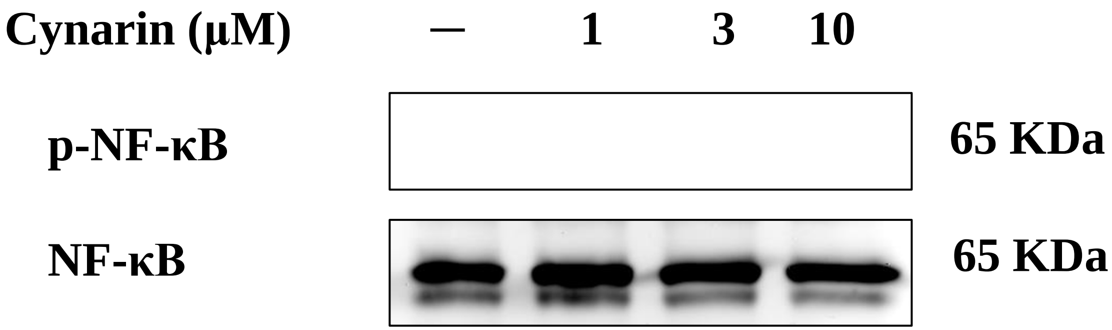
Cynarin (μM)
—
1
3
10
p-NF-κB
65 KDa
NF-κB
65 KDa

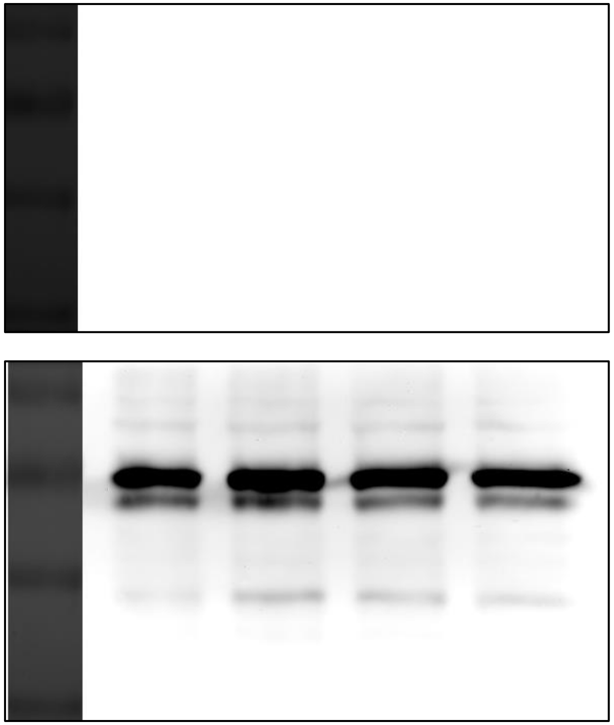

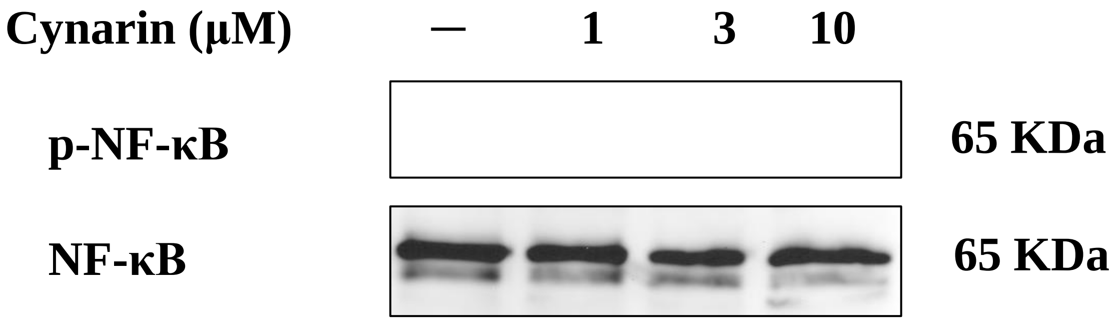
Cynarin (μM)
—
1
3
10
p-NF-κB
65 KDa
NF-κB
65 KDa

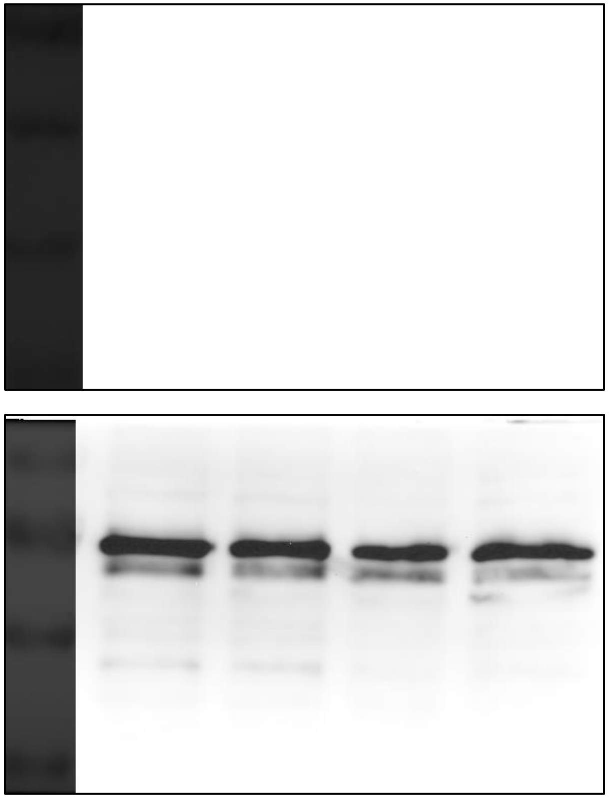

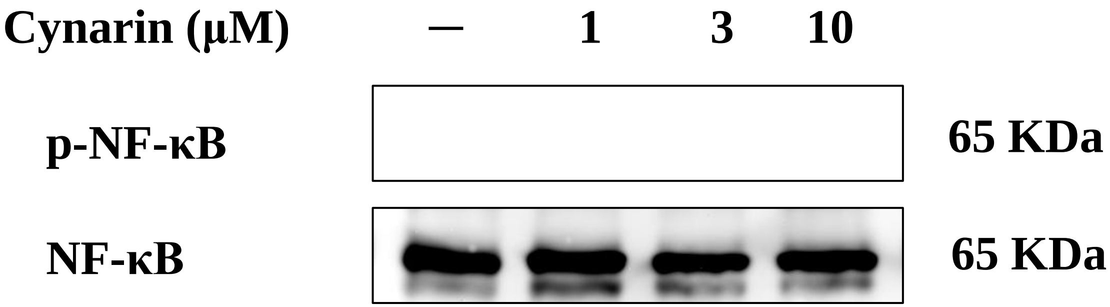
Cynarin (μM)
—
1
3
10
p-NF-κB
65 KDa
NF-κB
65 KDa

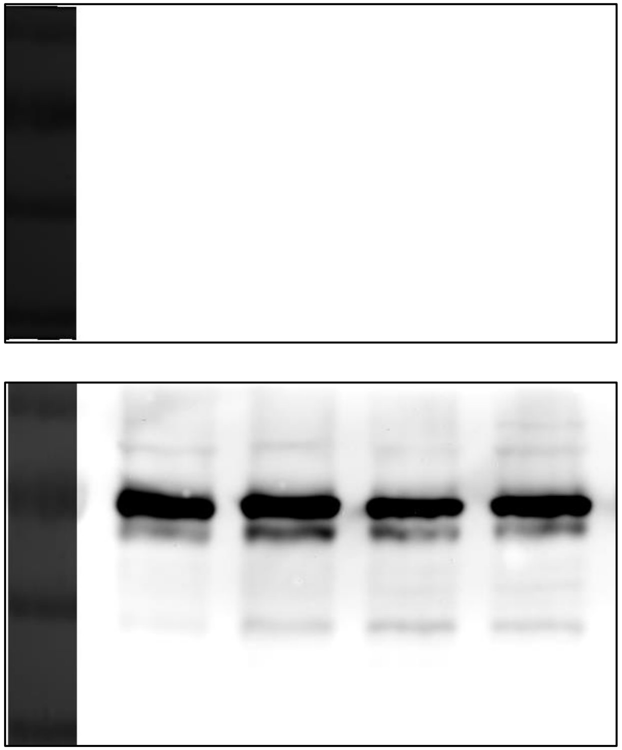

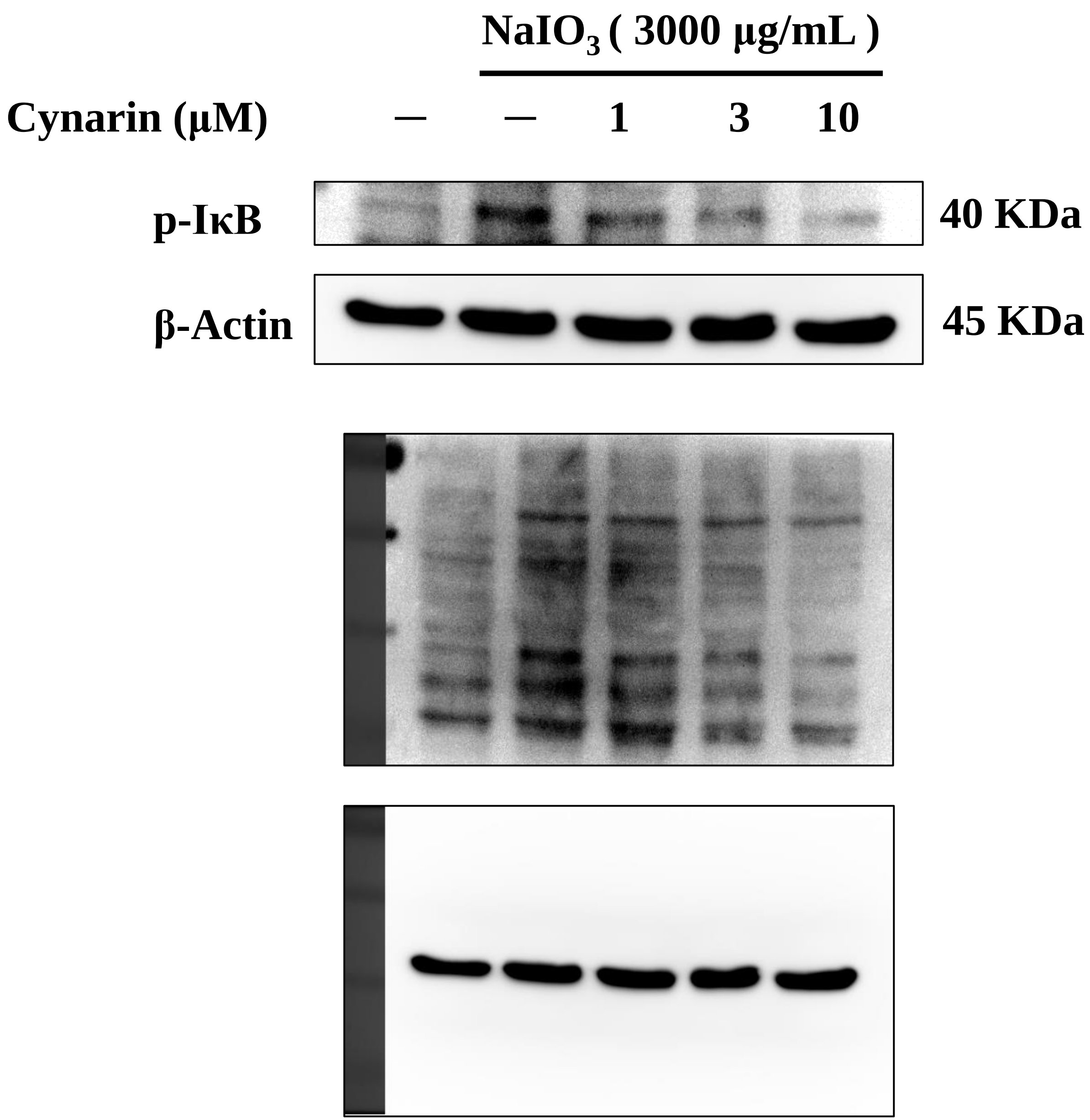
NaIO3 (3000 µg/mL)
Cynarin (µM)
—
—
1
3
10
p-IκB
40 KDa
β-Actin
45 KDa

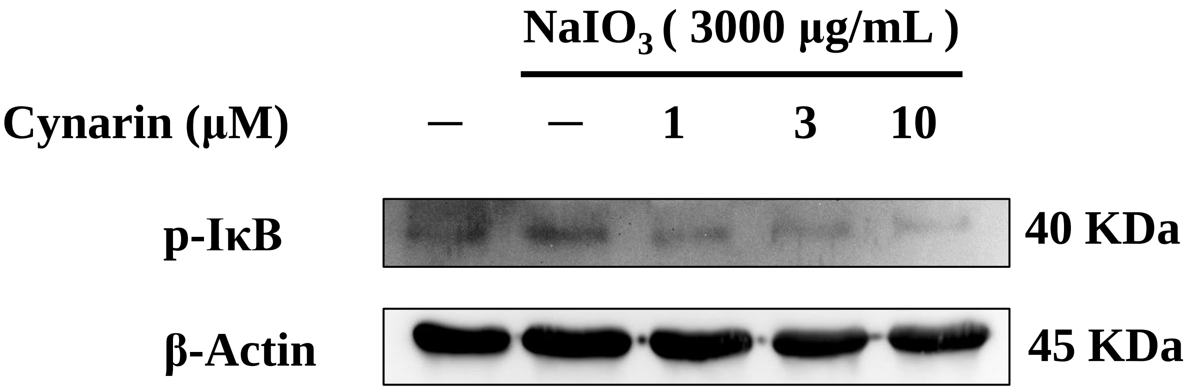

NaIO3 (3000 μg/mL)
Cynarin (μM)
—
—
1
3
10
p-IκB
40 KDa
β-Actin
45 KDa

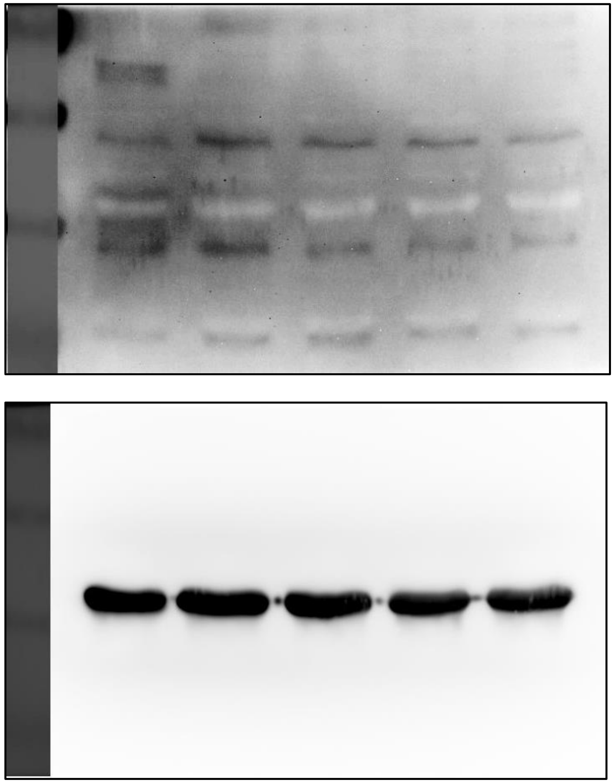

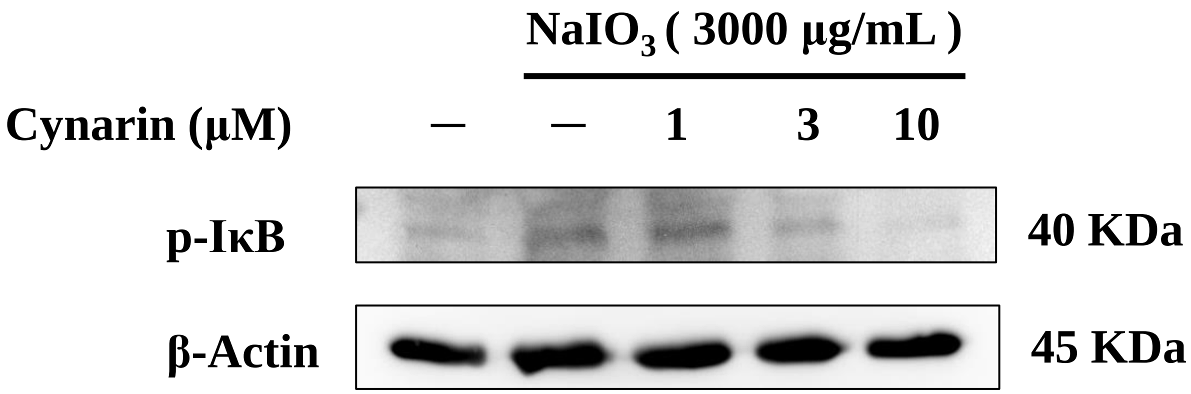

NaIO3 (3000 μg/mL)
Cynarin (μM)
—
—
1
3
10
p-IκB
40 KDa
β-Actin
45 KDa

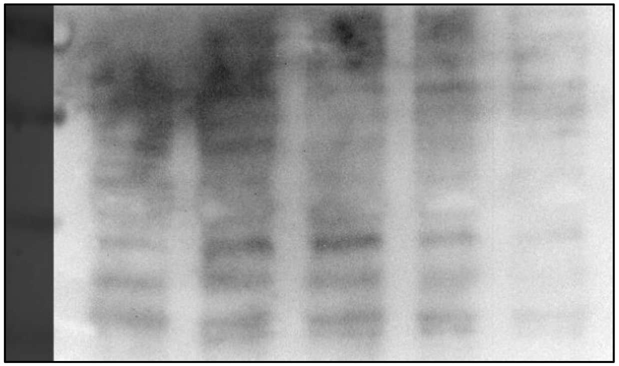

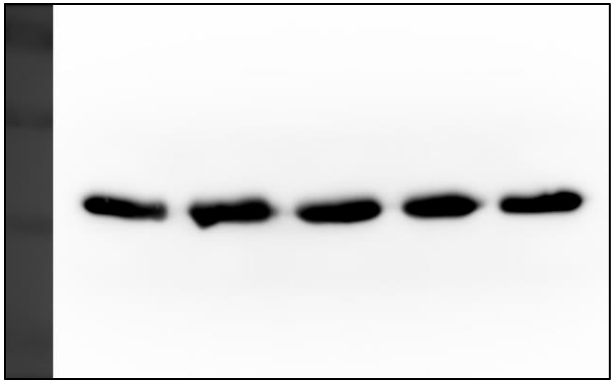

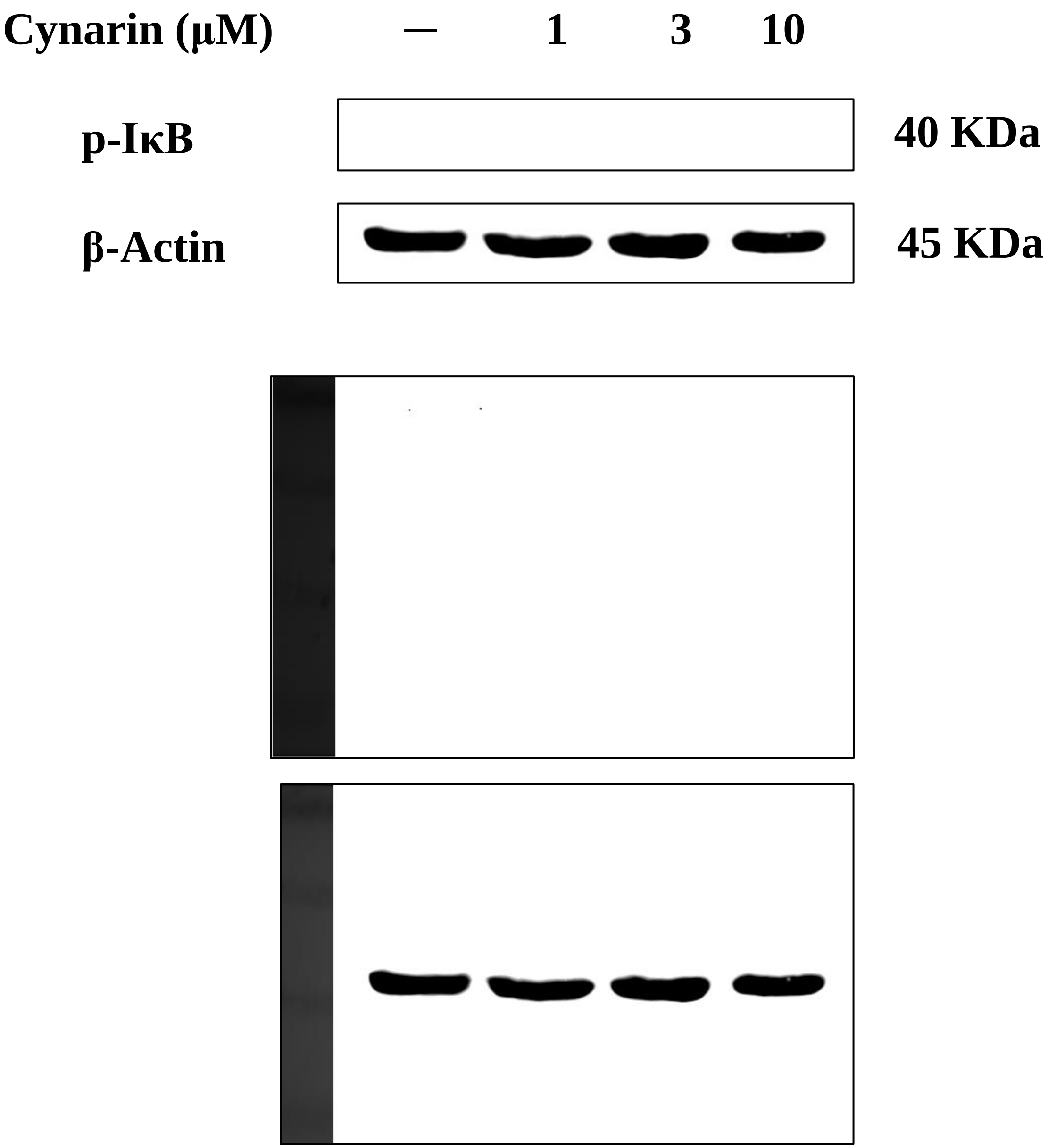
Cynarin (μM)
—
1
3
10
p-IκB
40 KDa
β-Actin
45 KDa

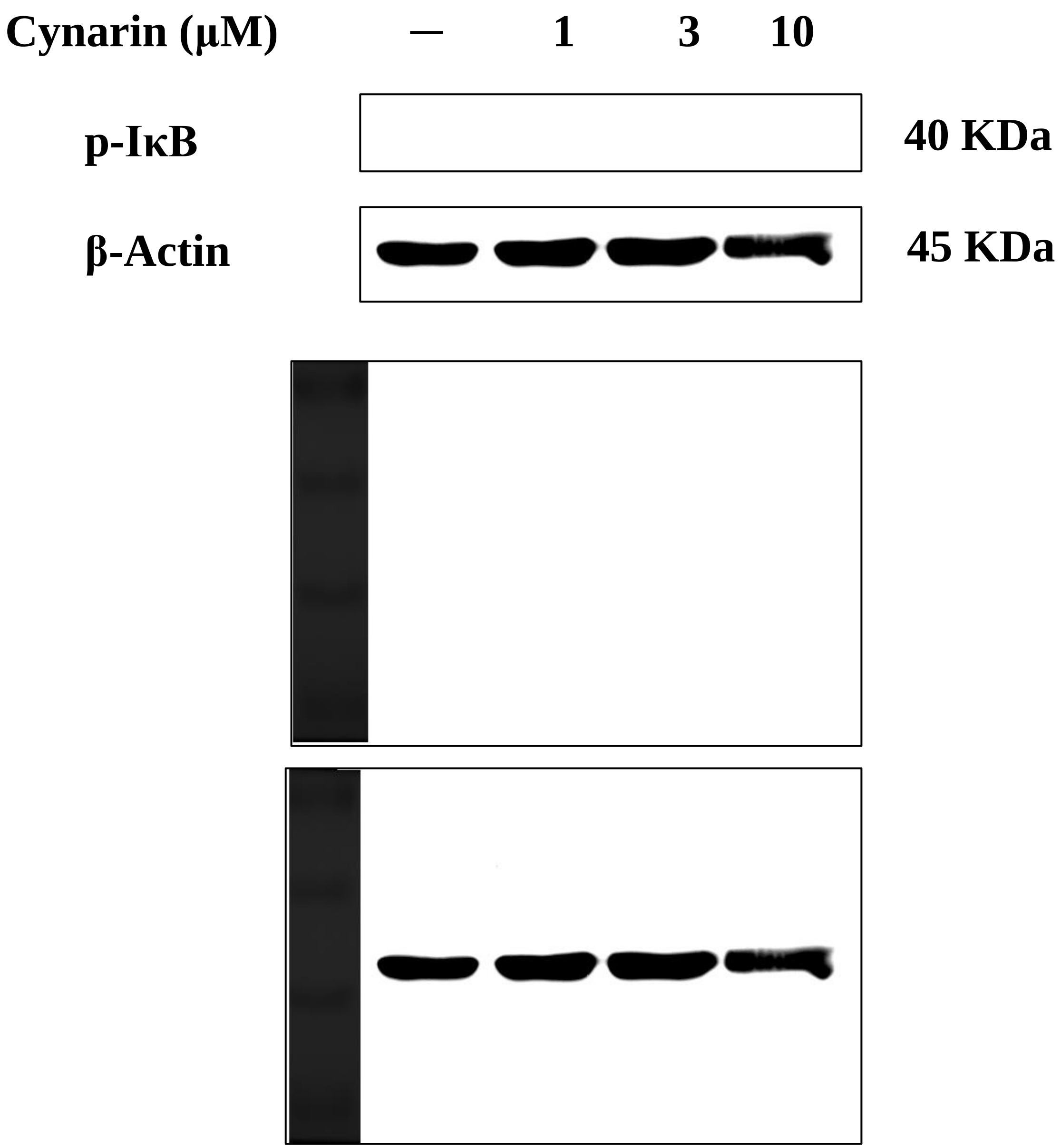
Cynarin (μM)
—
1
3
10
p-IκB
40 KDa
β-Actin
45 KDa

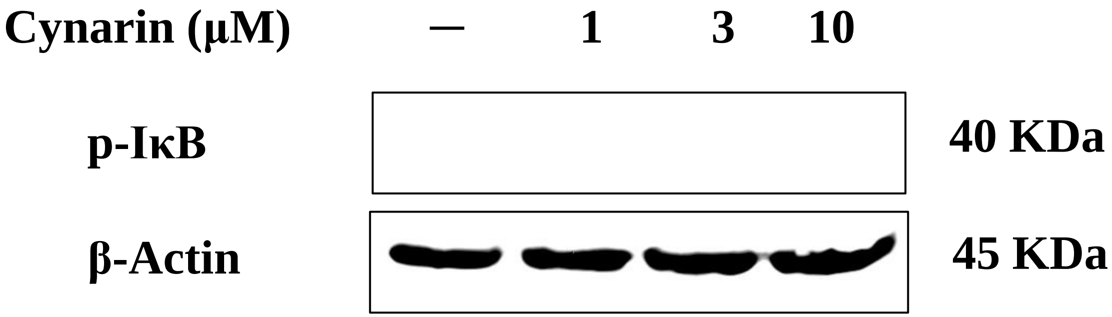
Cynarin (μM)
—
1
3
10
p-IκB
40 KDa
β-Actin
45 KDa

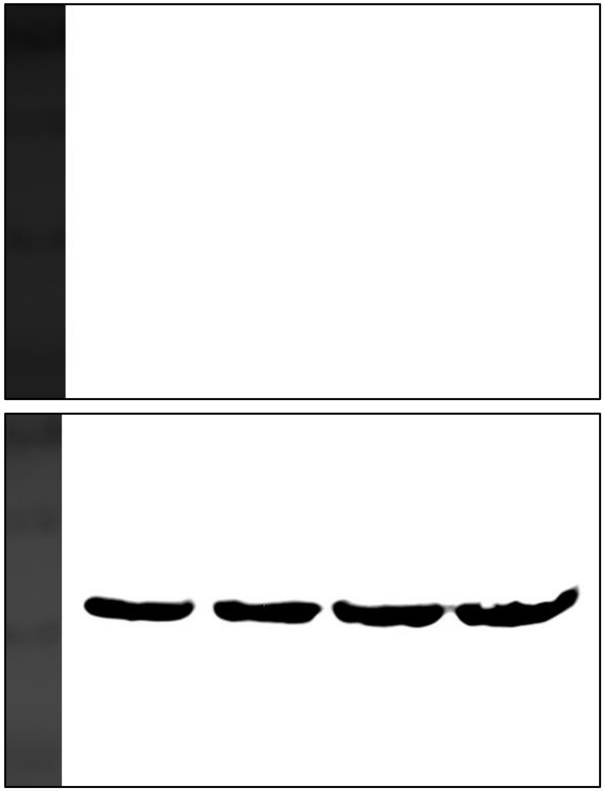

Supplement: Supplementary file 1 [file biomolecules-16-01227-s001.zip › biomolecules-4481888-supplementary.pdf]
